# Gut bacteria of adult and larval *Cotinis nitida* Linnaeus (Coleoptera: Scarabaeidae) demonstrate community differences according to respective life stage and gut region

**DOI:** 10.3389/fmicb.2023.1185661

**Published:** 2023-07-07

**Authors:** Roy A. Kucuk, Barbara J. Campbell, Nicholas J. Lyon, Emily A. Shelby, Michael S. Caterino

**Affiliations:** ^1^Department of Plant and Environmental Sciences, Clemson University, Clemson, SC, United States; ^2^Department of Biological Sciences, Clemson University, Clemson, SC, United States; ^3^National Center for Ecological Analysis and Synthesis, University of California, Santa Barbara, Santa Barbara, CA, United States; ^4^Department of Entomology, University of Georgia, Athens, GA, United States

**Keywords:** cetoniine, gut bacteria composition patterns, scarab beetle, gut microbiome, life stage analysis

## Abstract

The close association between bacteria and insect hosts has played an indispensable role in insect diversity and ecology. Thus, continued characterization of such insect-associated-microbial communities is imperative, especially those of saprophagous scarab beetles. The bacterial community of the digestive tract of adults and larvae of the cetoniine scarab species *Cotinis nitida* is characterized according to life stage, gut structure, and sex via high-throughput 16S rRNA gene amplicon sequencing. Through permutational ANOVAs of the resulting sequences, bacterial communities of the digestive system are shown to differ significantly between adults and larvae in taxon richness, evenness and relatedness. Significant bacterial community-level differences are also observed between the midgut and hindgut in adult beetles, while no significant host-sex differences are observed. The partitioning between bacterial communities in the larval digestive system is shown through significant differences in two distinct hindgut regions, the ileum and the expanded paunch, but not between the midgut and ileum portion of the hindgut region. These data further corroborate the hypothesis of strong community partitioning in the gut of members of the Scarabaeoidea, suggest hypotheses of physiological-digestive association, and also demonstrate the presence of a seemingly unusual non-scarab-associated taxon. These findings contribute to a general portrait of scarabaeoid digestive tract bacterial communities while illuminating the microbiome of a common new world cetoniine of the Gymnetini—a tribe largely neglected in scarab and beetle microbiome and symbiosis literature.

## Introduction

The insect digestive system is morphologically and physiologically dynamic and rich in bacteria and other microbes. The bacteria that comprise these gut communities can be symbiotic and correlate with the host’s diet, and in some cases contribute directly to host nutrient acquisition and other digestive-nutritive metabolic processes (Jing et al., 2020). An important first step in understanding the functionality, interspecific relationships, and ultimate evolutionary significance of the bacterial microbiome, as well as the potential applications, like medicine (Chevrette et al., 2019) and pest management (Qadri et al., 2020), is to determine the diversity and test for the presence of a “core community” within a host species, and to understand how this host-level community can be further delimited according the physiological and morphological variety of the gut. That is, knowledge of the presence of certain organisms at various taxonomic levels and of any predictable patterns relating to their presence enables more precise investigations of these organisms within the system, including the inference of their function with respect to the host.

The scarab beetles are a particularly speciose family of the Coleoptera whose members show a diversity in their diets (McKenna et al., 2019). Additionally, like many other holometabolous insects (insects exhibiting complete metamorphosis), the diets and feeding strategies of scarab beetles differ between adults and larvae, and this distinction is evident in life-stage differences in the alimentary canal (Shukla et al., 2016). Host-species-level diversity and corresponding dietary differences, as well as the differing diets between adults and larvae, the great morphological and physiological compartmentalization of the gut in general and the consequential remodeling of the digestive tract during metamorphosis present an opportunity for us to understand bacterial diversity and ultimately infer and test its function (Hammer and Moran, 2019).

Partitioning of bacterial communities is an apparent trend in scarabs, with each general region of the gut harboring its own distinctive community (Egert et al., 2003; Andert et al., 2010) including even host-species-unique members (Cazemier et al., 1999, 2003). Some studies have indicated that there are differences between the bacterial communities of adult and larval scarabs (Arias-Cordero et al., 2012; Shukla et al., 2016; Suárez-Moo et al., 2020), reflecting the differing habits, as well as varied routes of community transmission. Notably, this is even true among beetles with similar adult and larval diets, such as the dung beetles (Shukla et al., 2016). Indeed, differences can be checked by processes of transmission, which may involve communities passed down from the parents, thus ensuring similarity between developmental stages, as in the case of burying beetles for whom maternal care determines presence or absence of maternal gut bacteria (Wang and Rozen, 2017). While poorly studied, vertical acquisition of the gut microbiome, including members beneficial to their hosts, have been demonstrated in scarabs; for instance, the maternally transmitted community of a dung beetle has been described, and its presence is associated with enhanced development of larvae (Estes et al., 2013; Schwab et al., 2016). Thus, the bacterial microbiota of scarabs encapsulates many of the phenomena observed in such communities in the holometabolans, with a highly diverse assemblage, variable across “regions” of the digestive system (Arias-Cordero et al., 2012), and between life stages and sexes (Shukla et al., 2016). Additionally, in some cases it is vertically transmitted (Estes et al., 2013).

The cetoniine scarab, *Cotinis nitida,* or green June beetle, is a minor agricultural pest known to damage ripe fruit as an adult and turfgrass as a larva (Potter, 1991) and is widespread across the southeastern United States (Hammons et al., 2008). Like many other scarabs, and especially those of the Cetoniinae or “rose chafers,” it has an above-ground-dwelling imago with well-developed flight capabilities and a diet of sugar-rich foods (particularly ripe or decaying fruit), and a fossorial larval form that dwells, for the entirety of its development, in substrate rich in decaying plant material on which it feeds. Also like other scarabs, it exhibits a radically different adult and larval gut morphology: the adults possess a simplified digestive tract while the larvae have a gut noticeably subdivided into small foregut, midgut with three crowns of gastric caeca, and a hindgut divided into an ileum and a large baglike paunch.

Little is known of its microbiome, and what is known focuses mostly on a few microbial genera in limited contexts. For example, adult *C. nitida* beetles acquires its gut yeast flora after pupal eclosion (Vishniac and Johnson, 1990). In this gut yeast floral community, *Trichosporon cutaneum* is most abundant in the gut of adult beetle and has been shown to play a relatively important role in the behavioral biology of adult beetles by producing volatile semiochemicals that serve as aggregation pheromones (Vishniac and Johnson, 1990; Johnson and Vishniac, 1991). Similarly, a species of *Spiroplasma*, *S. clarkii*, has been cultured from the guts of both adult and larval *C. nitida* (Whitcomb et al., 1993). It is the aim of this work to identify the bacterial diversity of the green June beetle, and to test for community partitioning based on gut region, sex, and life stages in *C. nitida* through the analysis of 16S rRNA gene high-throughput amplicons. Specifically, we test whether the midgut and hindgut structures harbor communities of bacterial ASVs significantly different from each other. We also test if the bacterial microbiomes differ in adult beetles based on sex. Finally, we tested if bacterial microbiomes differ between larval and adult beetles.

## Materials and methods

### Sample collection

Adult beetles were collected after emerging from their pupal cells between June and September of 2018. With the exception of 5 individuals, which were not fed subsequent to capture, adults were placed in plastic containers and supplied with water-soaked paper towels and apple slices between capture and sample processing, which occurred when the beetles were still alive. Third instar larvae were collected from the soil between December of 2018 and February of 2019 and were also stored in plastic containers until processing. All individuals were collected from Clemson, South Carolina, USA. Adult *C. nitida* are distinguishable from all other scarabs in the region by their gross morphology—no other members of the genus or tribe are known from South Carolina, and the presence of a distinctive hood-like pronotum covering the scutellum distinguishes adults from vaguely similar species like *Euphoria fulgida. Cotinis* larvae are distinguished from those of other taxa by their size, manner of locomotion, and terminal setae. In total, 12 adults (seven males and five females) and 11 third instar larvae of indeterminate sex were sampled.

### Dissections

Guts were dissected from all individual specimens, which were dissected, alive, within 24 h of capture. Dissections were performed with 10% bleach-washed microscissors and forceps, in bleached glassware. Adult beetles were placed in a −20°C freezer for approximately 15 min and upon removal they were surface sterilized in 70% ethanol for one minute. They were then placed in a glass tray with approximately 20 mL of sterile phosphate buffered saline (PBS). Elytra were removed, as were wings. Microscissors were used to trim around the base of the spiracles and the tergites were pulled away, exposing the gut, tracheae, and fat body.

**Figure 1:**
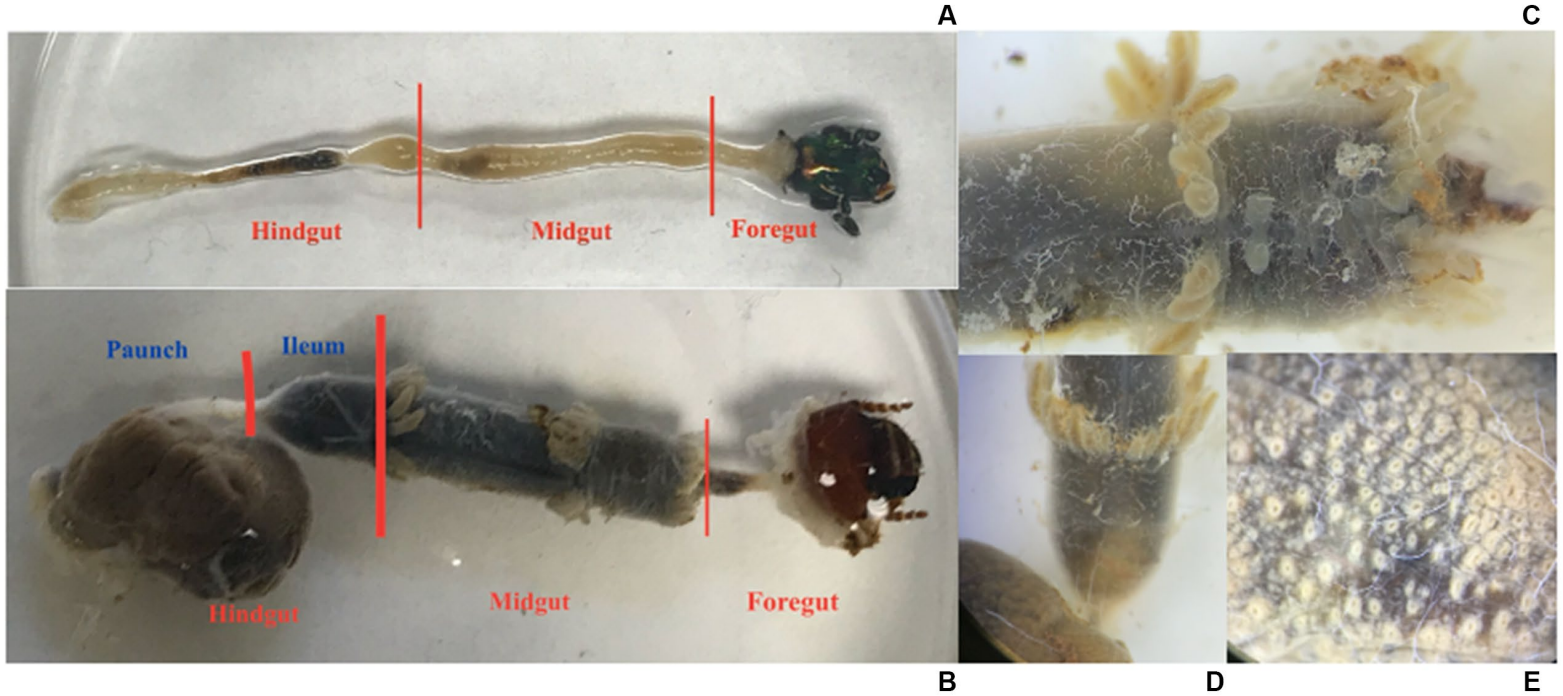
**(A)** The gut of an adult *C. nitida*, head to the right. **(B)** The gut of a larval *C. nitida*, head to the right. **(C)** Detail of the midgut of the larval *C. nitida*. The anterior portion of the gut midgut is facing the right. **(D)** Detail of the ileum of the larval *C. nitida*, which begins just below the posterior most crown of gastric caeca [where the Malpighian tubules arise, faintly visible below this crown in **(B)** and ends before the greatly expanded hindgut paunch to the bottom left]. Gastric caeca and the greatly expanded hindgut paunch to the bottom left. **(E)** Detail of the exterior paunch of the larval hindgut *C. nitida*, showing the off-white bases of papillae.

The prothorax was separated from the meso- and metathorax. The meso- and metathorax were removed by tearing out the tergum, pulling away any muscle or fat body tissue, and then pulling away the remaining structure delicately by the legs. The prothorax was removed by cutting the pleural region on either side of the pronotum to cut it in half, and both pieces were gently pulled away. To prevent lumen contents from leaking out of the midgut and small delicate foregut, the head was not removed. After using forceps to clamp the cuticle, a second pair of forceps were used to pull nervous, circulatory and respiratory tissue, fat body, and Malpighian tubules from the gut. The remainder of the beetle’s abdomen was pulled away from the rectum. The whole gut, including the foregut enclosed by the head, was moved to another sanitized tray with approximately 15 mL of sterile PBS. Microscissors were used to separate the hindgut from the midgut (Figure 1), and the midgut from the small foregut with the head.

Like adults, larvae were anaesthetized in a −20°C freezer 15 min prior to dissection and surface sterilized in 70% ethanol prior to being placed in sterile PBS. Incisions were made along the entire pleural region, below the spiracles. A circum-occipital cut was made, thus leaving a dorsal and ventral portion of the integument, which could be pulled apart and away from the head. Circulatory and respiratory tissue, fat body, and Malpighian tubules were pulled away from the gut. Any remaining cuticle was cut away from the around the anus. The gut, with the head still attached to the foregut, was transferred to a clean glass container filled with PBS. The gut was then cut into three sections: the paunch, the ileum, and the midgut (Figure 1). After dissection, all gut sections were immediately stored in a freezer at −80°C. The remains of adults and larvae not processed for extraction were stored in 100% ethanol. Given the partitioning, the adult guts amounted to 24 individual samples (12 midgut samples, and 12 hindgut samples), and the larvae amounted to 33 individual samples (11 midgut samples, 11 ileum samples, and 11 paunch samples). The remaining unused tissues from sampled individuals were saved as voucher specimens and deposited in the Clemson University Arthropod Collection.

### Extraction and test amplification

DNA from the gut sections of both adult and larval beetles were extracted using the DNeasy PowerSoil kit (QIAGEN Germantown). Given the large volume of liquid harbored by the larval gut sections and the potential to dilute reagents, these were first dehydrated in a vacuum centrifuge for 90 min. Both larval and adult gut sections were pulverized with sterile micropestles (Millipore Sigma St. Louis) prior to extraction. Extractions were carried out according to the protocol of the manufacturer, including suggested incubation steps. Extraction blanks were not used. Extracts were stored in a freezer at −20°C. Extracts were quantified using a Qubit 3.0 Fluorometer (Life Technologies Carlsbad). The 33 samples used had a DNA concentration above 1 ng/μl.

To test for potential inhibitory substances in the sample extracts, PCRs were prepared using 16.875 μl of nuclease-free water, 2.5 μl of Taq buffer, 0.125 μl of Taq polymerase, and 2.5 μl of dNTP mix (10 mM), 1 μl of forward and reverse primer for the V4 hypervariable region (10 μM), and 1 μl of DNA sample. For larval midgut samples, successful amplification required 6 μl of samples diluted to 1:100 of their original concentration. The settings used for the PCR were as follows: 94°C initial denaturation for 3 min (94°C denaturation for 20 s, 50°C annealing for 15 s, 72°C extension for 5 mins) × 25 cycles, 72°C final extension for 10 min, and 4°C incubation. These test runs were carried out on a Mastercycler nexus gradient (Eppendorf). Successful amplification was shown by the presence of a fluorescent band at the 400 bp mark and absence of primer-dimers. Foreguts of both adult and larval beetles were excluded from sequencing due to their small size, structural simplicity, and inability to yield bacterial DNA sufficient for amplification. For all PCRs, negative controls were used. The total number of individual samples suitable for sequencing was 49 (5 larval midgut samples, 11 ileum samples, 10 paunch samples, 12 adult hindgut samples, and 11 adult midgut samples).

### 16S rRNA gene amplification, library prep, normalization, and sequencing

Amplicon libraries were assembled using PCR to add sample-specific indexes and adapters to individual samples. Initially, we used 16.75 μl of nuclease-free water, 5 μl of buffer, 0.5 μl of dNTP mix, 2.5 μl of Phusion high fidelity DNA polymerase (New England BioLabs), 0.75 μl of forward and reverse primer, and 1 μl of DNA sample. Test runs were carried out on a C1000 Touch Thermal Cycler (Bio Rad) using Barcoded primers, 515F and 806R (Kozich et al., 2013; Apprill et al., 2015) for the amplification of the V4 hypervariable region to allow single-step multiplexed sequencing and reduce occurrence of chimeras (Callahan et al., 2019). For adult midgut, and adult hindgut samples, 6 and 4 μl of diluted (1:100) DNA extract was used for the PCR amplifications, respectively. The settings for most samples that yielded successful PCR product, without primer-dimers are as follows: 98°C initial denaturation for 3 min (98°C denaturation for 20 s, 61°C annealing for 15 s, 72°C extension for 5 min) × 25 cycles, 72°C final extension for 10 min, and 4°C incubation. The same protocol was used for the adult midgut samples, but 27 cycles were used to increase instances of amplification in these recalcitrant samples. Moreover, to better amplify larval midgut samples, KAPA HiFi DNA polymerase (KAPA Biosystems) was used instead, and the PCR was increased to 30 cycles. All gut samples that did not successfully amplify were omitted from the sequencing run. Consequently, only 4 out of the original 11 larval midgut samples and 11 of the original 12 adult midgut samples were kept. Normalization and library pooling was carried out according to established protocols (Kozich et al., 2013). Barcoded amplicons were sequenced on an in house MiSeq platform using the MiSeq Reagent Kit v2 (500 cycle) (Illumina) according to the protocol of the manufacturer.

### Data analysis

Analysis of 16 s rRNA gene sequences from *C. nitida* samples was carried out in QIIME 2 (version 2018.8; Bolyen et al., 2019). Sequences, as paired-end reads, were first joined using deblur (Amir et al., 2017). Quality filtering, including chimera removal and other denoising was carried out with deblur and representative sequences were generated using the command “representative-sequences.” Taxonomy was assigned with sequences from the SILVA database (version 132; Quast et al., 2013). Alignment of sequences was carried out using MAFFT (Katoh et al., 2002), followed by masking. Fastree (Price et al., 2010) was used to produce an unrooted tree, which was then rooted at the midpoint. To prevent the low frequency midgut samples of adult and larvae from being eliminated, the sampling depth was set at 1,000. This excluded 1 larval midgut sample from analysis on QIIME 2. The resulting sequences are henceforth referred to as amplicon sequence variants (ASVs), defined as different sequences recovered after the removal of erroneous elements generated by both PCR and subsequent amplicon sequencing. Community diversity analysis on QIIME was conducted for sex, gut region, and life stage with “alpha-group-significance” (for Faith’s PD) and “beta-group-significance” (for weighted and unweighted UniFrac, Bray–Curtis dissimilarity and Jaccard beta diversity analyses). To study the association between ASV and life stage or gut region, a multi-level pattern analysis was done using the package “indicspecies” (De Cáceres and Legendre, 2009), this package being chosen for its suitability in accounting for the compositional nature of the gut microbiome dataset (De Cáceres et al., 2012).

### Statistical analysis and visualization

Data tidying and diversity metric calculations used the qiime2R (version 0.99.2; Bisanz et al., 2018), remotes (Hester et al., 2021), “tidyverse,” “stringr” (version 1.3.1; Wickham and RStudio, 2019), “vegan” (version 2.6.0; Oksanen et al., 2020), and “fossil” (version 0.4.0; Vavrek, 2020) packages. Noting that a small number of the adult specimens were processed differently from the others during sample collection (3 adult hindgut samples and 2 adult midgut samples)–these not being provided with a food and water source during their capture–we excluded them from further analysis, leaving 43 suitable samples. We tested whether gut microbiome alpha or beta diversity differed across gut regions between adult and larval *C. nitida* with perMANOVAs (Anderson, 2017). Both permutation tests were run using the “lm.rrppp” function in the “RRPP” package in R (version 1.2.3; Collyer and Adams, 2021). Because some gut regions are not found in both life stages (i.e., the distinctly modified paunch, a development of the colon, is found in larvae only), a combination variable of life stage and gut region was used as the only explanatory variable. 10,000 permutations were used to reduce the variation inherent to permutation-based methods. Where the ‘stage-region’ was found to significantly affect a given response variable pairwise comparisons were run using the “pairwise” function from the “RRPP” package. Alpha diversity was tested using the following metrics: Chao1, ACE, Shannon diversity, Simpson diversity, Pielou’s evenness index, and Faith’s phylogenetic diversity. This was followed by beta diversity analysis, which used the entire community dataset though ordinations from Jaccard distance, Bray–Curtis dissimilarity, and both weighted and unweighted UniFrac distances (Lozupone et al., 2011). Additional analysis concerning ASV community structuring, namely relative abundance of families and genera at the level of life stage and gut region for the identification of indicator species associated with those regions was conducted using “indicspecies” (De Cáceres and Legendre, 2009).

Univariate metrics (i.e., alpha diversity) were visualized using the tidyverse package (version 1.3.1; Wickham and RStudio, 2021) while beta diversity was visualized with principal coordinates analysis (PCoA) using the “ape” package (Paradis et al., 2004). Figures displaying relative abundances at the phyla, family, and genera levels were visualized using the “ggplot2” package in R (Wickham, 2016). All tidying, analysis, and visualization of the data were performed in version 4.0.3 of the R statistical environment or QIIME2 (Bolyen et al., 2019; R Core Team, 2020). All data and R code will be made available via the version control platform “git” (Torvalds and Hamano, 2021) in a public repository following acceptance for publication (Lyon, 2021; Lyon, 2023).

## Data availability

The data can be found here: https://www.ncbi.nlm.nih.gov/bioproject/PRJNA931270.

## Results

### Overview of sequencing results

A total of 569,124 reads were obtained from 43 samples, yielding 3,622 bacterial and archaeal ASVs (Supplementary Table S1). These resulted in the identification of 285 genera, encompassing 23 phyla and 174 families, with only 2 and 6 of these, respectively, being archaeal.

### Alpha diversity

**Figure 2:**
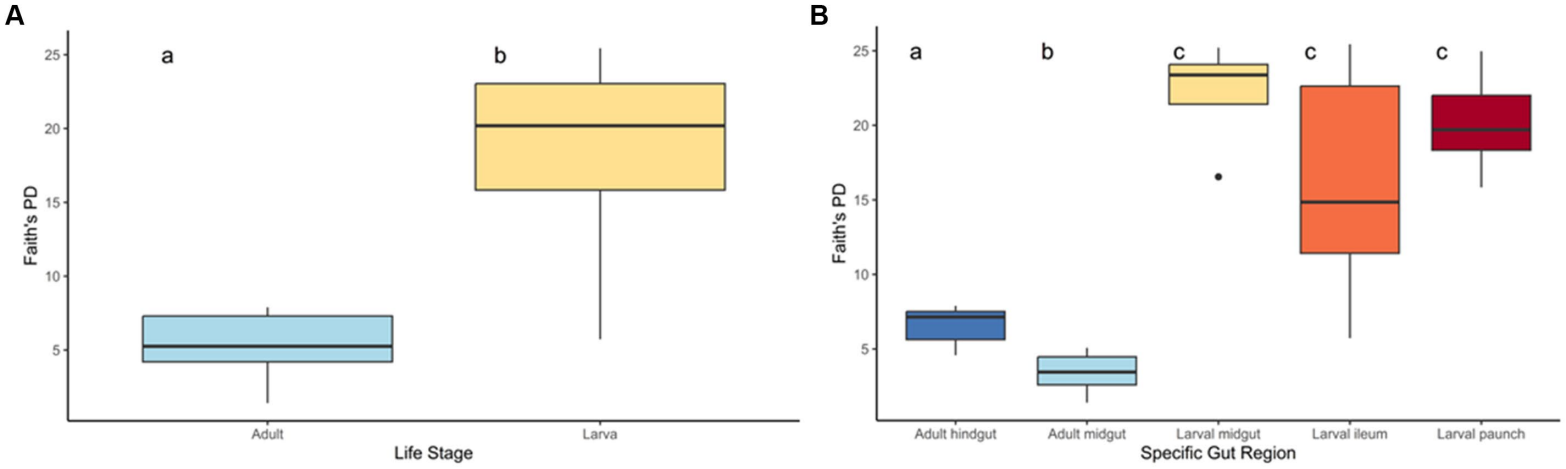
Phylogenetic diversity of the life stages **(A)** and gut regions **(B)** of *C. nitida*. The larval gut community consists of overwhelmingly more taxa than that of the adult. Letters **(a, b, c)** statistically significant distinctiveness.

The gut bacteria of adult and larval beetles showed a marked statistically significant difference in both phylogenetic diversity (Figure 2) and species richness and evenness (Supplementary Figure S1). Larval beetles harbored a taxonomically richer community of bacterial and archaeal ASVs than adults, according to Faith’s PD (H: 28.324696, *p* < 0.001, *q* < 0.001) (Figure 2). Additionally, the taxa present in larvae were distinct from those of adults (pseudo-*F*: 13.222026, *p* = 0.001, *q* = 0.001). Within adults, phylogenetic diversity of the midgut region was significantly lower from that of the hindgut (*H*: 12.595238, *p* < 0.001, *q* < 0.001). This distinction in phylogenetic diversity was not observed for the three regions of the larval that were sampled: the midgut was not significantly more diverse than the ileum (*H*: 2.454545, *p* = 0.117, *q* = 0.147) or the paunch (*H*: 1.28, *p* = 0.258, *q* = 0.287), and larval paunches were not significantly more diverse than ileums (*H*: 1.115702, *p* = 0.290846). In adult beetles, males and females did not harbor significant differences in the phylogenetic diversity of their gut bacteria (*H*: 0.46287, *p* = 0.496, *q* = 0.496).

This pattern was recapitulated in terms of evenness, as demonstrated by Shannon’s Evenness metric, with adults exhibiting less even communities than larvae (*F*-statistic: 23.4, *p* = 0.0001), adult midgut communities being less even than adult hindgut ones (*p* = 0.0414), larval midgut communities not differing significantly from larval ileums (*p* = 0.102) and paunches (*p* = 0.4422), and paunches not differing significantly from larval ileums (*p* = 0.2786). Differences in host sex did not significantly correlate with differences in evenness in terms of Shannon diversity (*F*: 0.1, *p* = 0.6975).

The above patterns were also seen in other measures of evenness (Simpson’s), as well as richness (Chao1 and ACE) (Tables 1, 2). That is, larval midgut, ileum, and paunch samples were consistently more diverse than those of adults. Moreover, paunch and midgut samples showed consistently high diversity. Variation was to be found in regard to adult midgut and adult hindgut differences, wherein adult hindguts exhibited more diverse communities than adult midguts according to metrics of phylogenetic diversity and evenness (Faith’s PD, Shannon’s, Simpson’s), and larval paunches exhibited more diverse communities than larval ileums in terms of richness (Chao1, ACE).

**Table 1:** Summary statistics of alpha and beta diversity analyses of *C. nitida* among life stage-gut region combinations and between sexes.

Explanatory variable	Response variable	Degrees of freedom	*F* statistic	Z score	*P-*value
Life stage and gut region	Chao1	4, 41	17.2	3.7	0.0001
	Simpson diversity	4, 41	17.4	4.0	0.0001
	Pielou’s evenness	4, 41	10.9	3.2	0.0001
	ACE	4, 41	17.3	3.7	0.0001
Sex	Chao1	1, 16	0.3	−0.2	0.5842
	Simpson diversity	1, 16	0.01	−1.4	0.9141
	Pielou’s evenness	1, 16	0.03	−1.2	0.8735
	ACE	1, 16	0.3	−0.2	0.5859

**Table 2:** Results of pairwise comparisons among gut regions for alpha and beta diversity.

Comparison	Chao1 P Values	Simpson Diversity P Values	Pielou’s Evenness *P* Values	ACE P Values	Community Matrix P Values
Adult hindgut v. Adult midgut	0.7087	0.0002*	0.0106*	0.7107	0.001*
Larval ileum v. Larval midgut	0.5757	0.3937	0.0073*	0.5687	0.998
Larval ileum v. Larval paunch	0.0413*	0.4322	0.6947	0.0409*	0.001*
Larval midgut v. Larval paunch	0.3542	0.8168	0.262	0.3592	0.037*

*P-*values with an asterisk (*) show statistically significant differences at *α =* 0.05.

### Beta diversity

**Figure 3:**
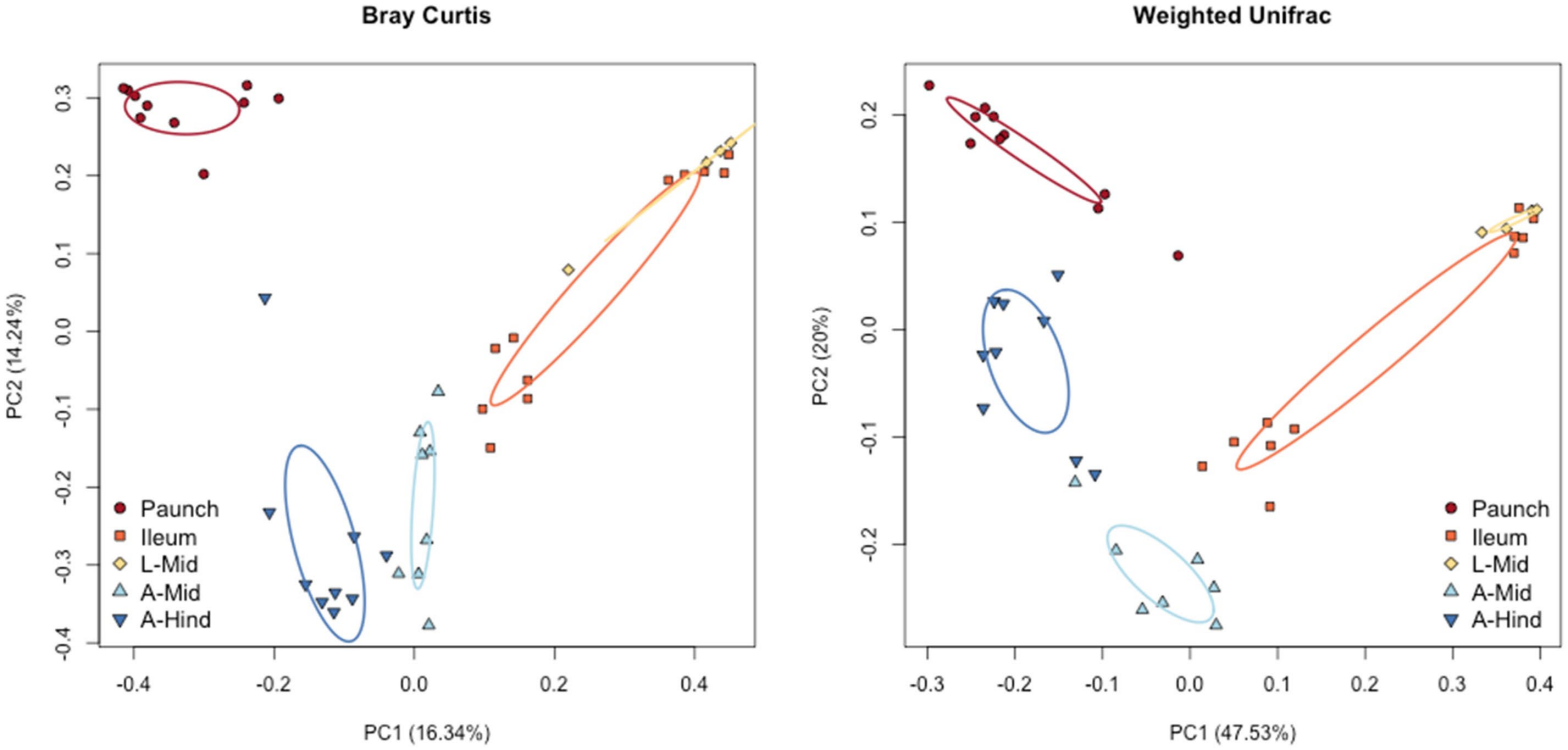
Beta diversity of the gut bacterial and archaeal communities of the sexes of *C. nitida* according to Bray–Curtis PcoA, and weighted UniFrac. The larval and adult communities are taxonomically distinct from one another, as are individual gut regions, particularly the larval paunch, midgut + ileum, and adult midgut and hindgut regions. Each dot represents the bacterial community of a single gut sample from a single beetle. Ellipses represent confidence regions.

**Figure 4:**
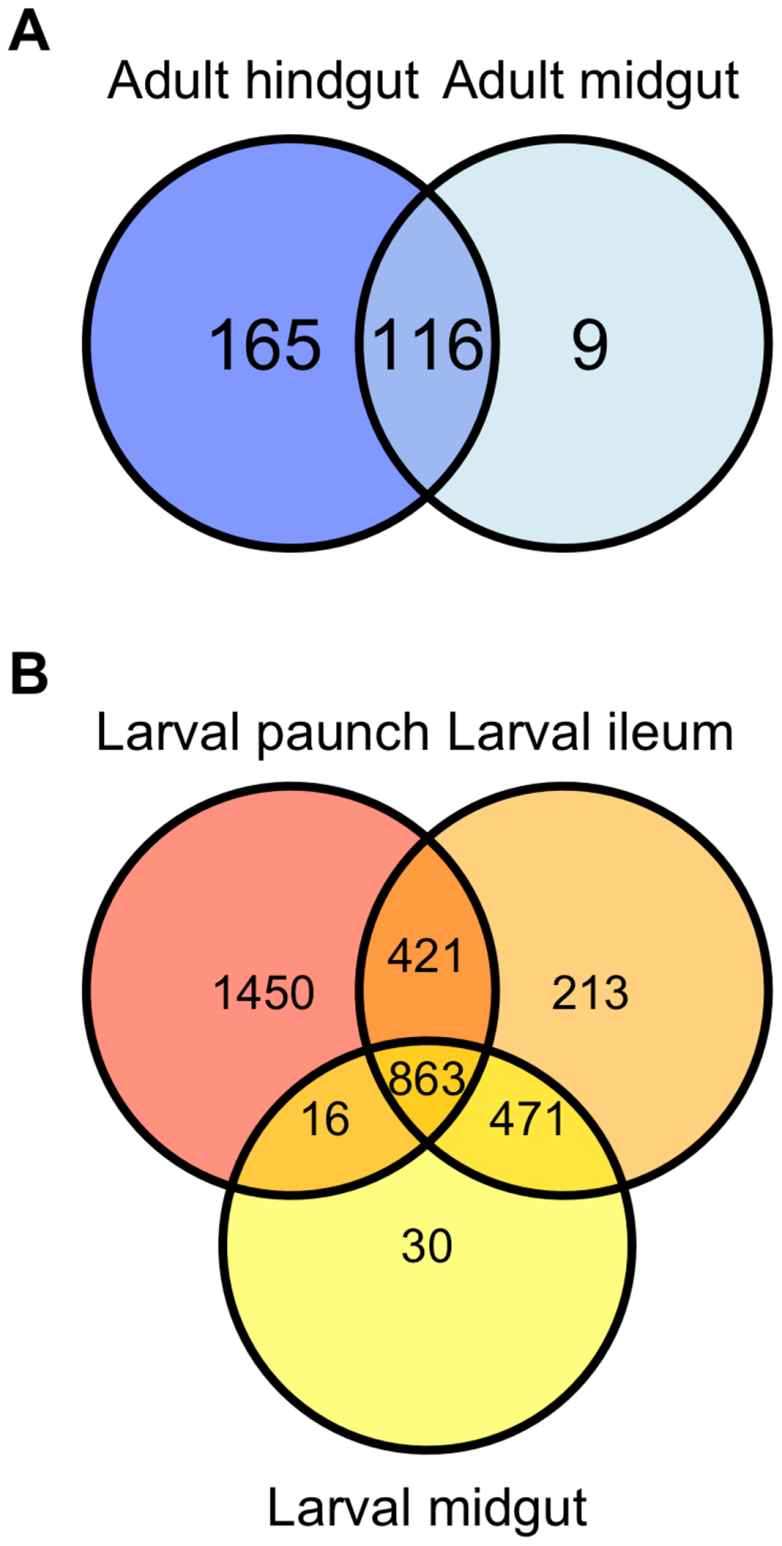
Venn diagram demonstrating overlap of community members (ASVs) in the gut of Adult **(A)** and larval **(B)** *C. nitida*.

Bray-Curtis and weighted UniFrac plots demonstrated a marked separation between adult and larval stages, as well as certain gut regions, namely between the adults and larvae and the hindgut paunch and other regions of the larval gut (Figure 3). Additionally, midgut samples grouped more closely with hindgut ileum samples from the same individual. Similar results were obtained in a Jaccard PCoA (Supplementary Figure S2). Unweighted UniFrac PCoA plots, in addition to demonstrating similar adult and larval differences, showed a distinctively grouped paunch community, as well as a larval midgut community that more closely resembles that of the larval ileum (Supplementary Figure S2). However, as corroborated by a perMANOVA, both models indicated an ileum community that differs from the other regions. Unweighted UniFrac PCoA plots also showed distinctive adult and larval communities as well as a distinction between adult and larval gut communities (Supplementary Figure S2). However, overlap between gut region community members does occur in both larvae adults (Figure 4).

Significant differences in community structure between life stages and gut regions, but not sexes, was found between *C. nitida* gut bacterial communities. There was a significant distinction between the adult midgut and adult hindgut (*F* statistic: 3.8, value of *p* = 0.001). However, there was no trend of taxonomic distinctiveness observed between the guts of male and female adult beetles (*F* statistic: 1.1, *p* = 0.327). All regions of the larval hindgut differed significantly from the regions of the adult gut. The larval midgut did not show significant taxonomic distinction with respect to the larval ileum (*F* statistic: 3.8, *p* = 0.998). However, the bacteria of the paunch showed a marked taxonomic distinction with respect to the midgut (*F* statistic: 3.8, *p* = 0.037) and the ileum (*F* statistic: 3.8, *p* = 0.001).

### Life stage and gut-region-specific abundance of bacterial phyla in *Cotinis nitida*

**Figure 5:**
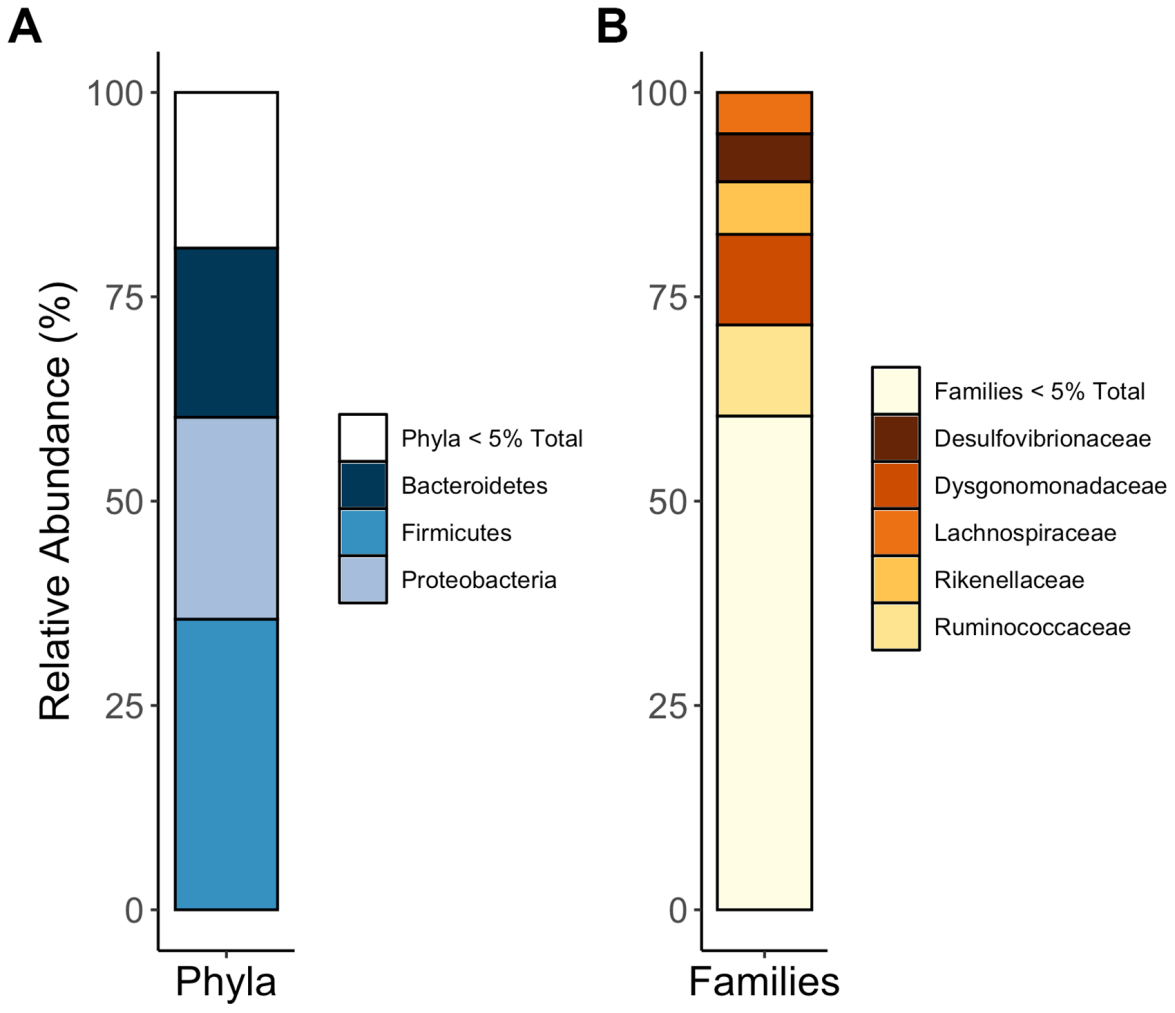
Phyla **(A)** and families **(B)** most abundant in the guts of *C. nitida*, indiscriminate of life stage, gut region, or sex.

**Figure 6:**
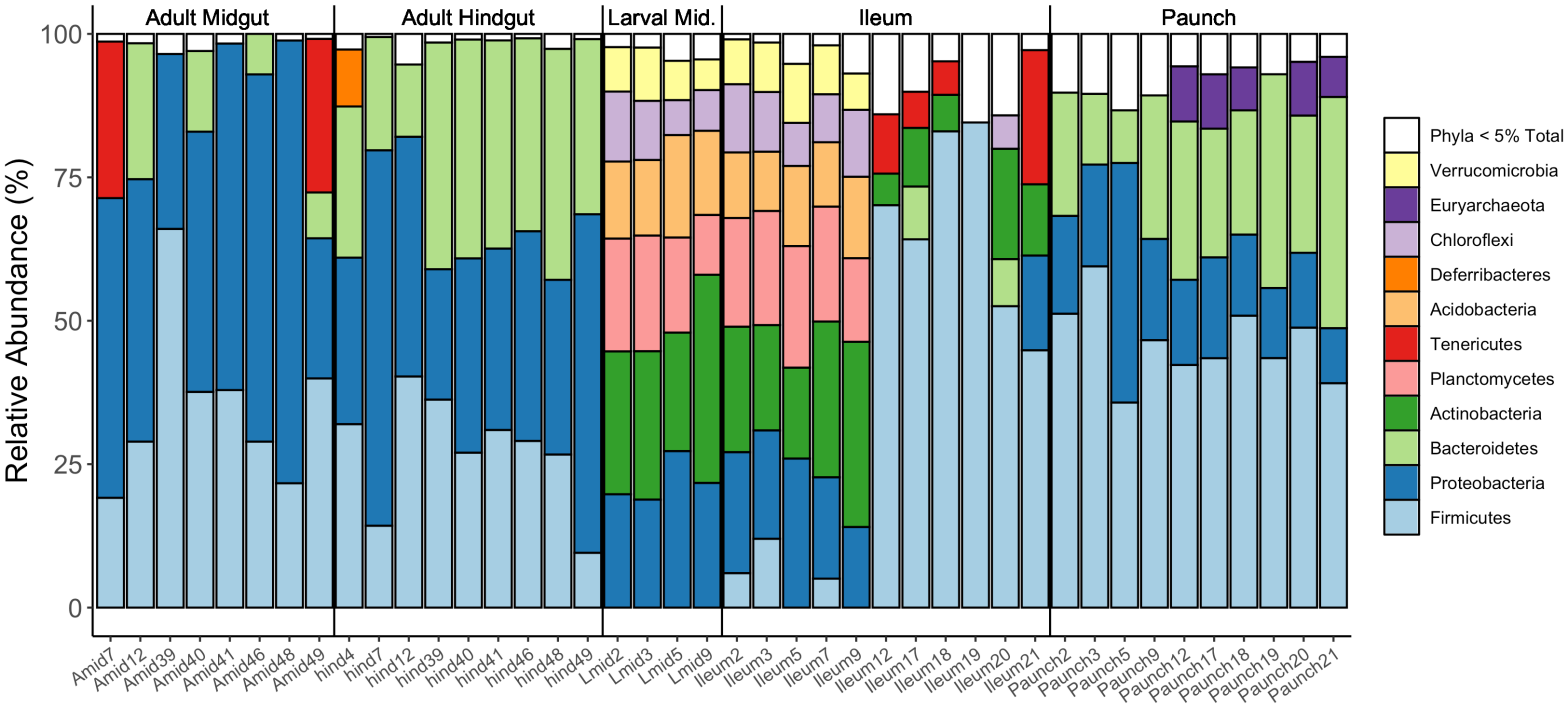
Relative abundances of bacterial phyla present in the various regions of the guts of adult and larval *C. nitida*. Only phyla of abundance levels of above 5% are shown here as distinct colored bars. Each column concerns an individual gut fragment (numbers following the gut fragment refer to the individual beetle the gut fragment was excised from).

**Figure 7:**
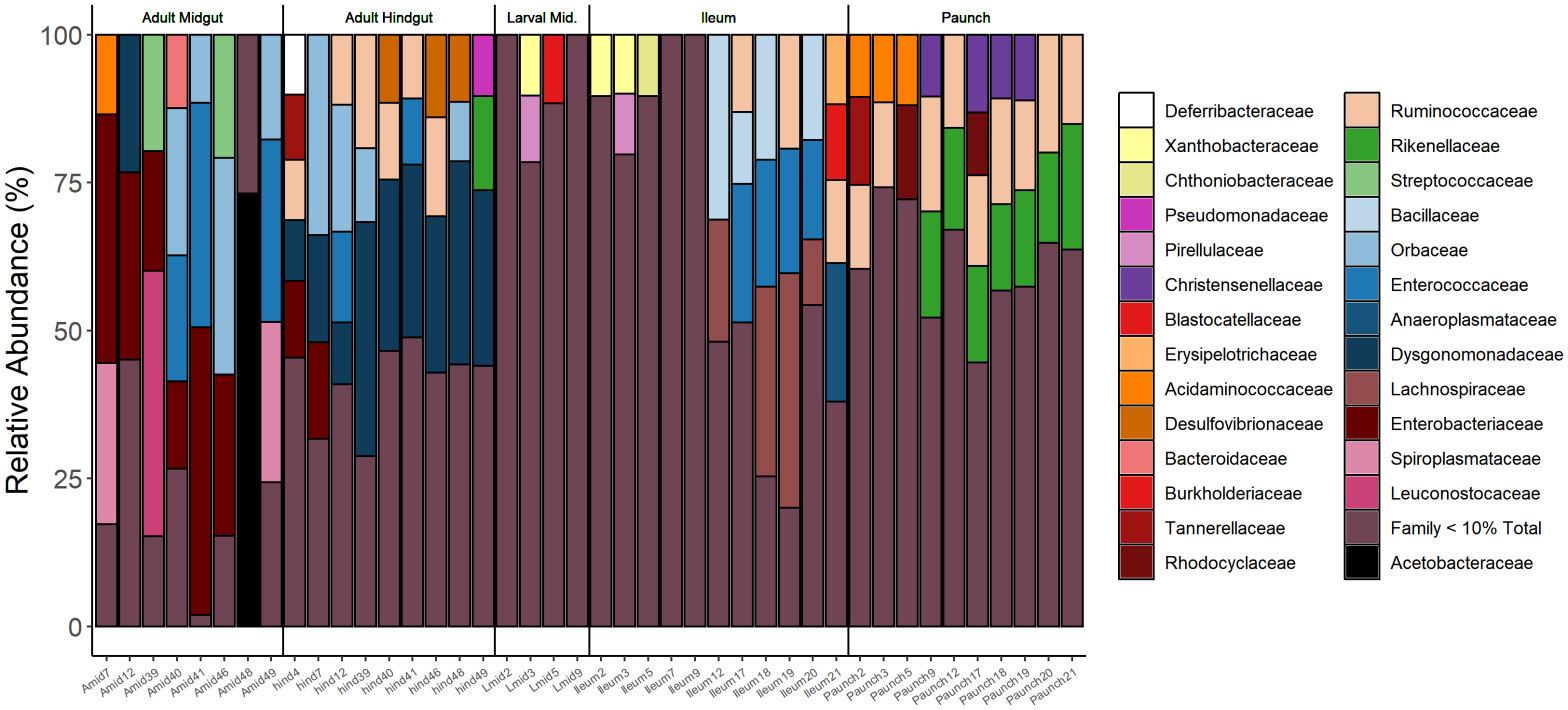
Relative abundance of bacterial families in the gut of adults (Midgut, Hindgut) and larvae (Midgut, Ileum, Paunch) of *C. nitida*. Families below 10% relative abundance have been excluded.

Together, the communities of adult and larval *C. nitida* are dominated by the bacterial phyla Firmicutes, Proteobacteria, and Bacteroidetes (Figure 5), while the most abundant families in *C. nitida* were Disulfovibrionaceae, Dysgonomonadaceae, Lachnospiraceae, Rikenellaceae, and Ruminococcaceae. However, the most dominant bacterial phyla, in terms of sheer abundance of representative ASVs, varied from one gut region type to the next (Figure 6), a situation also demonstrated by resident bacterial families (Figure 7). The larval midgut harbored communities dominated most heavily by Planctomycetes, followed by Verrucomicrobia, Actinobacteria, and Acidobacteria. The larval ileum of the hindgut harbored communities dominated by Firmicutes, followed by the Planctomycetes, Tenericutes, Verrucomicrobia, and Acidobacteria. The hindgut paunch of the larva differed sharply, with Bacteroidetes as the dominant phylum, followed by Firmicutes, the archaeal group Euarchaeota, and Deferribacteres. Adult beetles harbored Tenericutes as the most abundant midgut clade, followed by Proteobacteria, Firmicutes, and Bacteroidetes, while Bacteroidetes dominated the hindgut as with larvae, followed by Elusimicrobia, Proteobacteria, Firmicutes, and Deferribacteres.

### Life stage and gut-region-specific abundance of bacterial families in *Cotinis nitida*

Partitioning regarding abundance was observed in bacterial families present in the guts of both larval and adult *C. nitida*, with the families of greatest abundance (10% relative abundance or greater) in differing both between adults and larvae and between gut regions. However, with the exception of Dysgomonadaceae in the hindguts of adults, no given family-level taxon reached great abundance in all individuals of a particular life stage or gut type. However, a fraction of the most abundant taxa for each life stage or gut type were found in at least one third or more of the beetles sampled. Across sampled adults, these families were Desulfovibrionaceae, Dysgomonadaceae, Enterobacteraceae, Orbaceae, and Ruminococcaceae while in larvae the Bacillaceae, Christensenellaceae, Enterococcaceae, Lachnospiraceae, Rikenellaceae, Ruminococcaceae predominated. Regarding adult gut types, the adult midguts harbored Enterobacteraceae, Enterococcaceae, and Orbaceae, while the hindguts harbored Dysgomonadaceae, Orbaceae, Desulfovibrionaceae, Ruminococcaceae. In the larvae, the midguts harbored no taxa of great abundance in one-third or more the larvae, the ileums harbored Bacillaceae, Enterococcaceae, Lachnospiraceae, and over one-third of paunch samples harbored Christensenellaceae, Rikenellaceae, and Ruminococcaceae.

### Life stage and gut-region-specific abundance of bacterial genera in *Cotinis nitida*

**Figure 8:**
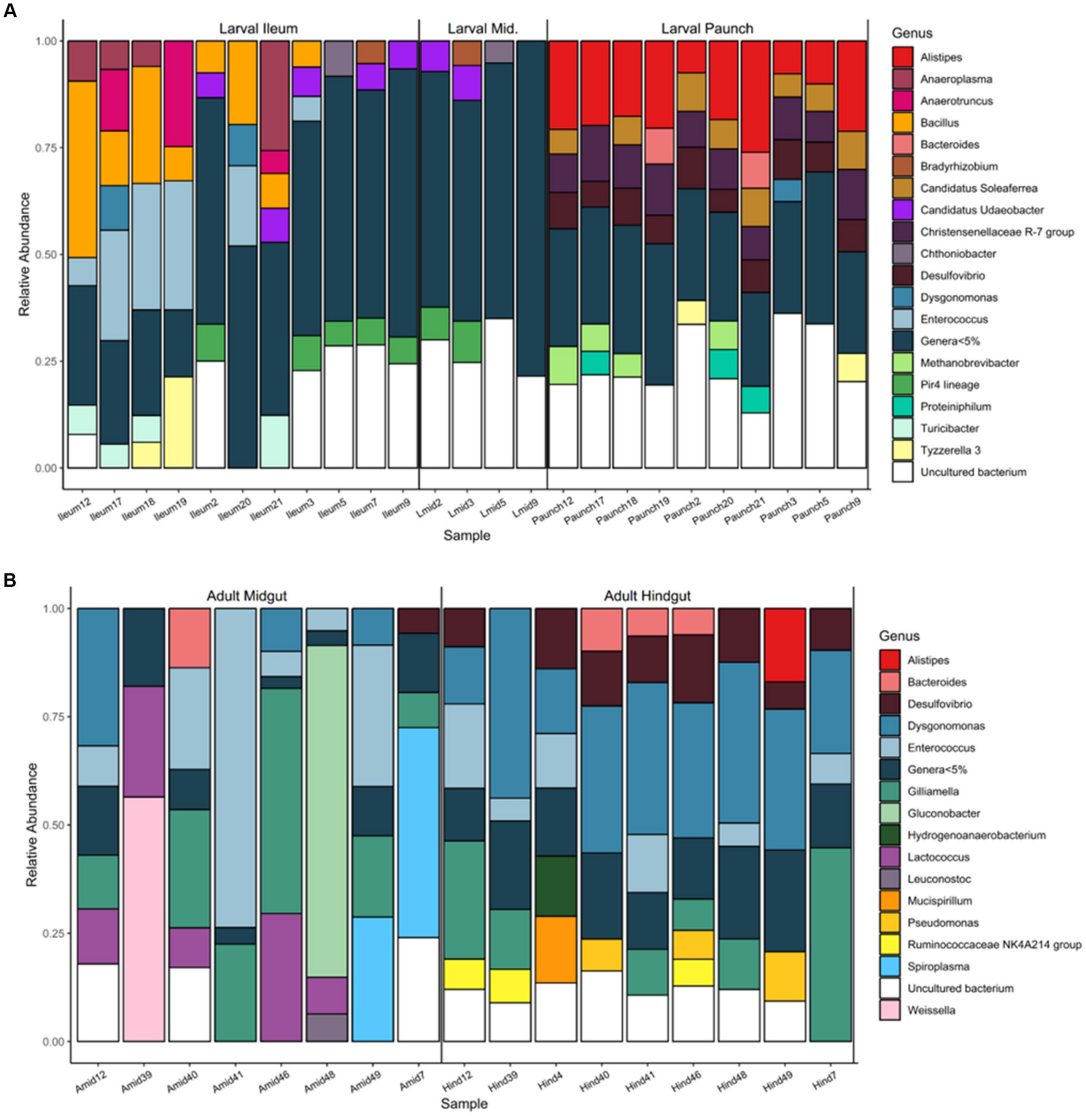
Abundance of bacterial genera found in the gut communities of larval **(A)** and adult **(B)** *C. nitida*, featuring taxa of 5% abundance or greater.

Partitioning in regard to abundant taxa was also seen in bacterial genera present in the guts of both larval and adult *C. nitida*, with the families of greatest abundance (here, 5% relative abundance or greater) differing both between adults and larvae and between gut regions (Figure 8). Unlike the case of bacterial family, more genera were found at great abundance in all individuals of a particular life stage or gut type, but this phenomenon, where present, was primarily exhibited in the larval paunch: genera *Alistipes*, Christensenellaceae of the R-7 group, and *Desulfovibrio* were found at high abundance in all paunch samples, while *Dysgonomonas* was found at high abundance in all adult hindgut samples. However, some taxa of comparatively great abundance were also present in at least 1/3 or more of samples. In the case of adults, this was true for *Desulfovibrio, Dysgonomonas, Enterococcus,* and *Gilliamella.* In the case of larvae, this was true for *Alistipes*, *Bacillus*, *Desulfovibrio,* Christensenellaceae of the R-7 group and Candidatus *Soleaferrea.*

Within specific gut regions, some genera were abundant in 1/3 or more of samples as well (Figure 8). In adult midguts, this was the case with *Enterococcus*, *Dysgonomonas Gilliamella*, and *Lactococcus*. In adult hindguts, such taxa were represented by *Desulfovibrio*, *Bacteroides*, *Dysgonomonas*, *Enterococcus*, *Gilliamella*, and NK4A214 group Ruminococcaceae. In the comparatively few larval midguts sampled, *Udaeobacter* and Bacteria of the Pir4 lineage were most abundant in 1/3 or more of samples, while in the ileums this was the case for *Anaeroplasma*, *Bacillus, Enterrococcus, Udaeobacter*, and Bacteria of the Pir4 lineage. Finally, in larval paunches, bacteria of the genus *Alistipes*, Christensenellaceae of the R7 group*, Desulfovibrio*, *Methanobrevibacter*, and Candidatus *Soleaferrea* were found in 1/3 or more of samples.

### Life stage and gut-region-specific bacterial indicator families of *Cotinis nitida*

The guts of *C. nitida* on the level of life stage and gut region also bear region-specific families of indicator taxa (Table 3). The indicator taxa of each region differed from each other in terms of number as well, with the larvae having a far greater assemblage of indicator families than adults. In both larvae and the adults, these indicator taxa increased posteriorly, with more indicator taxa present in the hindgut than the midgut of adults and likewise in the larvae, with more indicator taxa in the hindgut than the midgut and more such taxa in the paunch than the ileum. Adult indicator families are represented by members of the phyla Firmicutes and Proteobacteria, which are also represented among larval indicator families in addition to 10 other larval indicator phyla (Acidobacteroidota, Actinomycetota, Chloroflexi, Euarchaeota, Gemmatimonadota, Nitrososphaerota, Planctomycetota, Tectomicrobia, and Verrucomicrobiota). Adult midgut families are represented solely by the phylum Firmicutes, while adult hindgut indicator families are represented by the phyla Proteobacteria and Thermodesulfobacteriota. Larval midgut indicator families belong to Bacteroidetes and Proteobacteria, while Ileum indicator families belong to Bacteroidetes and four other phyla (Firmicutes, Tenericutes, Proteobacteria, and Verrucomicrobiota), and the paunch indicator families consisted of the phyla Firmicutes, Bacteroidetes, Euryarchaeota, Gracilibacteria, and Proteobacteria. The indicator families did not overlap much with the most abundant families: in adults only three of the 17 most abundant taxa were indicator taxa (Leuconostocaceae, Orbaceae, Streptococcaceae), while in larvae, whose indicator taxa were represented by 47 families, only six of the 16 most abundant families were also indicator taxa (Bacillaceae, Blastocatellaceae, Cthonobacteraceae, Christensenellaceae, Pirellulaceae, and Xanthobacteraceae). In specific gut regions, only one adult midgut -abundant taxon (Streptococcaceae) and one hindgut taxon (Orbaceae) were also indicator taxa, and in larvae no midgut indicator taxa were abundant, while one ileum taxon (Anaeroplasmataceae) and two paunch taxa (Rhodocyclaceae, Ruminococcaceae) were indicator taxa.

**Table 3:** Family level taxa of each life stage and gut region of *C. nitida.*

Life stage	Family	Stat	*P*-value
Adult gut	Enterobacteriaceae	0.605	0.001
	Streptococcaceae	0.482	0.001
	Orbaceae	0.439	0.001
	Lachnospiraceae	0.346	0.001
	Leuconostocaceae	0.31	0.001
	Leuconostocaceae	0.278	0.005
Larval gut	Micrococcaceae	0.532	0.001
	Promicromonosporaceae	0.519	0.001
	Steroidobacteraceae	0.502	0.002
	Iamiaceae	0.487	0.001
	Acidothermaceae	0.479	0.001
	Pirellulaceae	0.472	0.001
	Gemmatimonadaceae	0.472	0.001
	Nitrososphaeraceae	0.47	0.001
	Hyphomicrobiaceae	0.47	0.001
	Rhodomicrobiaceae	0.459	0.002
	Paenibacillaceae	0.452	0.001
	Mycobacteriaceae	0.451	0.001
	Ktedonobacteraceae	0.449	0.001
	Xanthobacteraceae	0.449	0.002
	Syntrophomonadaceae	0.448	0.003
	Solirubrobacteraceae	0.447	0.001
	Christensenellaceae	0.447	0.004
	Bacillaceae	0.446	0.001
	Rhodobacteraceae	0.445	0.002
	Alicyclobacillaceae	0.443	0.002
	Verrucomicrobiaceae	0.436	0.001
	Streptomycetaceae	0.433	0.001
	Gemmataceae	0.428	0.002
	Ilumatobacteraceae	0.426	0.002
	Opitutaceae	0.425	0.005
	Xiphinematobacteraceae	0.425	0.002
	Methanosaetaceae	0.414	0.004
	Nocardioidaceae	0.414	0.001
	Gaiellaceae	0.411	0.001
	Intrasporangiaceae	0.411	0.001
	Rubinisphaeraceae	0.408	0.002
	Chthoniobacteraceae	0.408	0.002
	Peptococcaceae	0.408	0.005
	Streptosporangiaceae	0.393	0.001
	Methyloligellaceae	0.391	0.005
	Micromonosporaceae	0.391	0.001
	Thermomonosporaceae	0.37	0.001
	Devosiaceae	0.356	0.001
	Pyrinomonadaceae	0.351	0.002
	Reyranellaceae	0.348	0.005
	Roseiflexaceae	0.347	0.003
	Blastocatellaceae	0.343	0.003
	Caldilineaceae	0.338	0.003
	Entotheonellaceae	0.337	0.003
	Isosphaeraceae	0.328	0.002
	Microbacteriaceae	0.289	0.001
	Sphingomonadaceae	0.289	0.002
Adult midgut	Streptococcaceae	0.628	0.005
Adult hindgut	Enterobacteriaceae	0.686	0.003
	Beijerinckiaceae	0.655	0.003
	Desulfobacteraceae	0.633	0.003
	Orbaceae	0.61	0.002
Larval midgut	Dongiaceae	0.712	0.001
	Microscillaceae	0.576	0.005
Larval ileum	Bacillaceae	0.712	0.001
	Methyloligellaceae	0.651	0.002
	Anaeroplasmataceae	0.636	0.003
Larval paunch	Christensenellaceae	0.9	0.001
	Rikenellaceae	0.893	0.001
	Peptococcaceae	0.88	0.001
	Opitutaceae	0.869	0.001
	Syntrophomonadaceae	0.845	0.001
	Ruminococcaceae	0.821	0.001
	Methanosaetaceae	0.82	0.002
	Clostridiaceae	0.726	0.003
	Flavobacteriaceae	0.661	0.004
	Rhodocyclaceae	0.658	0.002
	Burkholderiaceae	0.653	0.001
	Gracilibacteraceae	0.652	0.004

### Life stage and gut-region-specific bacterial indicator genera of *Cotinis nitida*

At the genus level, indicator taxa were most numerous in larvae, consisting of 135 different genera, and particularly in the larval paunch and ileum (45 genera and 38 genera, respectively), but were also observed in the larval midgut and in the adult hindgut (just four genera and eight genera, respectively). Few of these were also particularly abundant genera, however, In terms of abundance with overlap, two out of the four most abundant taxa present in adults (*Gilliamella* and *Dysgonomonas*) were also indicator taxa of adult guts. In the larval gut, a single highly abundant taxon (R-7 group Christensenellaceae) out of six others was also represented among the numerous indicator taxa. The larval midgut did not have any overlap between its most abundant taxa and its indicator taxa. In larval ileums, a single abundant genus (*Bacillus*) out of five was also an indicator genus. In the larval paunch, by contrast, abundant genera were more well-represented among indicator taxa among the five most abundant taxa present, four (R-7 group Christensenellaceae, *Alistipes*, *Methanobrevibacter*, and *Soleaferrea*) were also indicators. In both adult hindguts and larval paunches was a shared indicator genus, *Desulfovibrio*, which was also one of the most abundant and widespread genera in *C. nitida* overall.

## Discussion

This study characterized the gut community of adult and larval members of the cetoniine scarab beetle *C. nitida*. Diversity analyses revealed that *C. nitida* not only possesses a taxonomically rich community of bacteria, but that this richness varies markedly between both adults and larvae and between gut regions within the same life stage. Gut community composition did not significantly differ between adult females and adult males. We observed patterns including gut-morphology-centered community partitioning that largely corroborate the general portrait of scarabaeoid beetle gut communities. Understanding these patterns helps to not only make predictions about the composition of gut communities in other organisms, but also to understand potential functions of the microbial communities along the digestive tract.

### Bacterial community diversity of the gut of *Cotinis nitida* and hypotheses of localization

Our hypothesis regarding gut bacterial community differences based on life stage was confirmed. Not only did larvae possess more diverse communities phylogenetically (over 11 times more ASVs in their digestive tract than adults), but their microbial communities were also more diverse in terms of evenness and richness. Larva also harbored distinct taxa. Overall, this community partitioning resembles that found in other scarabaeoids (Wang et al., 2020).

The absence of a significant difference between male and female beetles in both alpha and beta diversity is markedly different from other scarab beetles, notably the dung beetles (Shukla et al., 2016). However, this discrepancy could be due to parental behavior differences between the two species. Because female scarab beetles are the sole parent participating in larval feeding, larvae develop a gut bacterial community that is more similar to the female than the male (Shukla et al., 2016). *C. nitida* does not exhibit parental care, and, therefore, would not be expected to have a gut bacterial community more like one parent than another.

Additionally, the hypothesis regarding bacterial community diversity based on gut region was also confirmed. Within adults, the bacterial communities of the midgut and hindgut differed in terms of phylogenetic diversity and evenness, with the hindgut being more phylogenetically diverse and even. Within larvae, the paunch had a distinct community signature. The larval ileum and midgut showed more similarities in community dynamics. Several occurrences could explain this phenomenon. This less stringent grouping of communities may indicate a far more transient community in the midgut and ileum. For example, the wide passage of the anterior ileum and the physiological similarities between this region and the anterior midgut may naturally facilitate a community crossover between the midgut and ileum. The poor clustering of ileum samples suggests a less consistent community of organisms compared to that of the paunch. It is possible that the ileum simply shares taxa by virtue of several physiological and morphological conditions of the beetle gut, including but not limited to condition of ingested substrate, gut pH, oxygen level, host digestive enzymes, and host immune response, as seen in other insect taxa (Maire et al., 2018; Lampert et al., 2019). Additionally, the presence or absence of certain taxa may be influenced by the microbial community itself. It is possible that hindgut paunch physiology favors some taxa over others. For example, oxygen content can influence community makeup, i.e., the lack of oxygen promotes the domination of anerobic bacteria such as Christensenellacae and Methanosaetacae (Chen and He, 2015; Kropp et al., 2021). Such patterns are observed in other animals, including some other insects (Engel and Moran, 2013), and indicates settlement of bacterial communities along the digestive tract is not a consequence of ingestion, but rather that underlying patterns relating to the host’s environment and specific selection processes are at play. The lack of statistically significant overlap between bacterial communities of the paunch, which was more morphologically delimited from the other two segments and thus less prone to disturbance during the dissection process further bolsters this hypothesis. Moreover, morphology should also be considered when hypothesizing about community localization, for the paunch is separated from the ileum by a greatly constricted length of hindgut. It is also internally lined with papillae, the basis of which can be observed through the translucent colon, and whose facilitation of retaining digested material can be hypothesized to affect residential microbial consortia. However, the crossover between taxa found in the larval ileum and midgut may be indicative of human error regarding dissection approach and gut morphology. Although the gut is filled with large pieces of plant material that do not move readily, lumen fluid can easily shift from one portion of the gut to another during dissection. Though, the notion that dissection error is solely responsible for these results can be overturned when considering the clustering of ileum samples with midguts from the same individual, compared to ileums from other individuals.

While mirroring most trends observed in other insects, the differences in taxon richness and community partitioning based on gut region and life stage in *C. nitida* is emblematic of what is known of the scarab gut microbiome. However, as stated previously, the lack of significant difference in taxon richness between midgut and hindgut regions of larvae in this study differs from that of other scarabs (Andert et al., 2010; Arias-Cordero et al., 2012). The distinctiveness of bacterial communities according to measures of alpha and beta diversity regarding life stage and individual gut region is not unprecedented, and other insects, including scarab beetles, exhibit similar patterns of bacterial diversity (Egert et al., 2003). This is also the case for other animals that have highly complex, compartmentalized digestive systems, including ruminating mammals and humans (Albenberg et al., 2014; Donaldson et al., 2016). Moreover, many relatively abundant bacterial families in the guts of *C. nitida* have representatives associated with particular physiological processes in the digestive systems of other organisms including Ruminococcaceae, Lachnospiraceae, Desulfovibrionaceae, Rikenellaceae (abundant in the hindguts of adults and the hindguts of larvae, although most abundant in the paunch) with cellulolytic activity in the digestive systems of other scarabs as well as ungulates (Biddle et al., 2013; Handique et al., 2017; Alonso-Pernas et al., 2017a,b; La Reau and Suen, 2018; Wang et al., 2022), Bacillaceae (abundant in the ileums of larvae) with iron reduction in the midguts of other scarabs (Hobbie et al., 2012), Burkholderiaceae (abundant in all regions of the larval gut, but most abundant in the hindgut paunch) and Orbaceae (abundant in adult midguts and hindguts) with nutritional symbiosis, respectively, in other insects (Kikuchi et al., 2012; Zheng et al., 2016), and Spiroplasmataceae (abundant in the adult midgut) with defensive symbiosis and reproductive manipulation in other insects (Xie et al., 2014).

### Bacterial genera and their hypothetical roles in the guts of adult and larval *Cotinis nitida*

In terms of most abundant and region-indicative genera of larval *C. nitida*, we find numerous taxa that are generally expected of animal digestive tracts. Among the most prominent of these “usual suspects” can be found in the larval paunch. For example, the R-7 group of the Christensenellaceae has been characterized as a volatile fatty acid producer (Goodrich et al., 2014), as well as a digestor of complex carbohydrates and amino acids, respectively converting these into acetate and ammonia (Chen et al., 2020). *Alistipes*, in addition to being potentially influential member of human guts (Parker et al., 2020), is well-represented in fungus-growing termites (Schnorr et al., 2019), suggesting a role as a gut symbiont in general. The paunch-centric Candidatus *Soleaferrea*, which also appears in the guts of numerous organisms, from mammals (Cai et al., 2020) to insects (McManus et al., 2018), has been found in hindguts of scarabs, and is noted for a fermentative habit (Ebert et al., 2021)—a habit that befits the environmental conditions of the scarab hindgut (Andert et al., 2010; Huang and Zhang, 2013; Ebert et al., 2021). *Desulfovibrio*, an anaerobic sulfate-reducer, is yet another common gut resident in animal taxa (Rey et al., 2013; Holman et al., 2022), including non-scarab insects (Mikaelyan et al., 2016), but in particular has been demonstrated to colonize the same region in the hindguts of scarabs of the subfamilies Melolonthinae and Scarabaeinae (Egert et al., 2005; Huang and Zhang, 2013; Ebert et al., 2021) at similarly great levels of abundance, further bolstering a gut-centric habit and sulfate-reducing service in *C. nitida*. *Methanobrevibacter*, an anaerobic methanogen (Enzmann et al., 2018), was also detected from the hindguts of a melolonthine scarab, further suggesting a broader preference for this specific anatomical region on the part of anaerobes (Egert et al., 2005).

In the distinctive larval ileum, the genus *Bacillus* was one of the few genera of over 5% abundance to be found in more than 1/3 of samples, mirroring overall significant presence in larval guts, but not specific region localization–this genus in particular is associated with iron reduction in *midguts* of larval scarabs (Hobbie et al., 2012), rather than hindguts. However, this may indeed may be a consequence of inclusion of the larval ileum with the midgut, which calls attention to possible level of gut localization that may be occurring, even in seemingly uniform or similar structures. In ileums as well as some larval midguts we also see *Udaeobacter*, a genus common in soil (Willms et al., 2020), which suggests a relationship between general larval habitat and the organisms that colonize the gut. This is also true of the Pir4 lineage (also present in the midguts of larvae); a group known for its anaerobic associations, including the habitation of low-oxygen soil (Dedysh et al., 2020).

In adult beetles, few of the most abundant taxa were found in more than 1/3 of the samples, the exceptional cases being *Gilliamella*, *Dysgonomonas*, and, as in larvae, the genus *Desulfovibrio*. With the exception of *Gilliamella*, the most abundant of these were restricted to the hindgut of adults, suggesting, as in larvae, a more selective hindgut community at the generic level. As in the cases of larvae, the adult beetles possess among their most abundant and widespread residents generally animal-gut-associated taxa like *Dysgonomonas*, which, while found in various terrestrial animals, including humans (Hofstad et al., 2000) has known insect associations, including residence in the guts of termites (Pramono et al., 2015) as well as other cetoniine scarabs (Schroeder et al., 2022). The most intriguing of the widespread and abundant genera in adult guts was the genus *Gilliamella*. *Gilliamella* of the Orbaceae, known as a bee symbiont, is a particularly unprecedented finding, although recent work, describing the genus and its close relatives from brachyceran flies (Jose et al., 2021; Hong et al., 2022) and tenebrionid beetles (Huang et al., 2019; Kuo et al., 2021), suggests a more extensive presence in insects, although not necessarily a mutualistic one.

### Limitations and future directions

Overall, whether or not the relatedness of certain taxa to studied organisms with similar gut localizations and cooccurrences with other microbial taxa reflects any manner of functional similarity is tenuous; at best the suggestion of such similarities provides a basis for future hypothesis testing. Additionally, the great diversity of *C. nitida* gut communities, particularly in larvae, makes functional hypothesizing weak without at least metagenomic scrutiny, as well as thorough culturing and proteomics approaches.

Additional factors which were not considered for the present study include the relationship between age of the sampled organisms (time since eclosion for either larvae or adults) and ag -related physiological processes. Though metamorphosis ceases after the imago stage, feeding behavior, as well as certain physiological shifts, incumbent on maturation may alter the bacterial community profile. Other limitations of this study include location of sampling, host diet variation, and small sample size. The gut communities of some scarabs change across instars (Alonso-Pernas et al., 2017a,b; Chouaia et al., 2019), as well as the provenance of taxa that appear to be common or abundant across one gut region or another, may be influenced by shifts in host diet or the geographic location from which the individual was sampled—our samples were taken from a very small geographical area, and thus their gut communities may reflect microhabitat in addition to broader geographical associations. Future work should seek to test how this occurs in *C. nitida* and to ultimately test for uniformities of microbial community structure in the scarabaeoids overall. Nevertheless, this study adds to the growing body of literature on gut bacterial community dynamics in *C. nitida.*

## Data availability statement

The datasets presented in this study can be found in online repositories. The names of the repository/repositories and accession number(s) can be found at: https://www.ncbi.nlm.nih.gov/, PRJNA931270.

## Author contributions

RK: project design, sample collection, data analysis, and manuscript preparation. BC: methodological guidance and manuscript drafting. NL: data analysis, writing (methodology), and public repository provisioning. ES: statistical analysis and figure generation. MC: project design, methodological guidance, and manuscript preparation. All authors contributed to the article and approved the submitted version.

## Funding

This publication was supported by the John and Suzanne Morse Endowment for Arthropod Biodiversity.

## Conflict of interest

The authors declare that the research was conducted in the absence of any commercial or financial relationships that could be construed as a potential conflict of interest.

## Publisher’s note

All claims expressed in this article are solely those of the authors and do not necessarily represent those of their affiliated organizations, or those of the publisher, the editors and the reviewers. Any product that may be evaluated in this article, or claim that may be made by its manufacturer, is not guaranteed or endorsed by the publisher.

## Supplementary material

The Supplementary material for this article can be found online at: https://www.frontiersin.org/articles/10.3389/fmicb.2023.1185661/full#supplementary-material

## References

[ref1] Albenberg L. Esipova T. V. Judge C. P. Bittinger K. Chen J. Laughlin A.. (2014). Correlation between intraluminal oxygen gradient and radial partitioning of intestinal microbiota. Gastroenterology 147, 1055–63.e8. doi: 10.1053/j.gastro.2014.07.020, PMID: 25046162 PMC4252572

[ref2] Alonso-Pernas P. Arias-Cordero E. Novoselov A. Ebert C. Rybak J. Kaltenpoth M.. (2017a). Bacterial community and PHB-accumulating Bacteria associated with the wall and specialized niches of the hindgut of the Forest cockchafer (*Melolontha hippocastani*). Front. Microbiol. 8:291. doi: 10.3389/fmicb.2017.00291 28293223 PMC5329036

[ref3] Alonso-Pernas P. Bartram S. Arias-Cordero E. M. Novoselov A. L. Halty-deLeon L. Shao Y.. (2017b). *In vivo* isotopic labeling of symbiotic Bacteria involved in cellulose degradation and nitrogen recycling within the gut of the Forest cockchafer (*Melolontha hippocastani*). Front. Microbiol. 8:1970. doi: 10.3389/fmicb.2017.01970, PMID: 29075241 PMC5643479

[ref007] Amir A. McDonald D. Navas-Molina J. A. Kopylova E. Morton J. T. Zech X. Z.. (2017). Deblur Rapidly Resolves Single-Nucleotide Community Sequence Patterns. mSystems 2, e00191–e00116. 28289731 10.1128/mSystems.00191-16 PMC5340863

[ref4] Anderson M. J. (2017). “Permutational multivariate analysis of variance (PERMANOVA)” in Wiley StatsRef: statistics reference online. eds. Balakrishnan N. Colton T. Everitt B. Piegorsch W. Ruggeri F. Teugels J. L.

[ref003] Andert J. Marten A. Brandl R. Brune A. (2010). Inter- and intraspecific comparison of the bacterial assemblages in the hindgut of humivorous scarab beetle larvae (Pachnoda spp.). FEMS Microbiol Ecol. 74, 439–449. 20738398 10.1111/j.1574-6941.2010.00950.x

[ref5] Apprill A. McNally S. Parsons R. Weber L. (2015). Minor revision to v4 region rRNA 806R gene primer greatly increases detection of SAR11 bacterioplankton. Aquat. Microb. Ecol. 75, 129–137. doi: 10.3354/ame01753

[ref004] Arias-Cordero E. Ping L. Reichwald K. Delb H. Platzer M. Boland W. (2012). Comparative evaluation of the gut microbiota associated with the below- and above-ground life stages (larvae and beetles) of the forest cockchafer. Melolontha hippocastani. PLoS One. 7:e51557. 23251574 10.1371/journal.pone.0051557 PMC3519724

[ref8] Biddle A. Stewart L. Blanchard J. Leschine S. (2013). Untangling the genetic basis of Fibrolytic specialization by Lachnospiraceae and Ruminococcaceae in diverse gut communities. Diversity 5, 627–640. doi: 10.3390/d5030627

[ref009] Bisanz J. E. (2018). qiime2R: Importing QIIME2 artifacts and associated data into R sessions. Version 2.

[ref006] Bolyen E. Rideout J. R. Dillon M. R. Bokulich N. A. Abnet C. C. Al-Ghalith G. A.. (2019). Reproducible, interactive, scalable and extensible microbiome data science using QIIME 2. Nat Biotechnol. 37, 852–857. Erratum in: Nat Biotechnol. 37:1091. 31341288 10.1038/s41587-019-0209-9 PMC7015180

[ref9] Cai J. Zhou L. Song X. Yin M. Liang G. Xu H.. (2020). Alteration of intestinal microbiota in 3-deoxyglucosone-induced Prediabetic rats. Biomed. Res. Int. 2020:8406846. doi: 10.1155/2020/8406846 32908918 PMC7468600

[ref005] Callahan B. J. Wong J. Heiner C. Oh S. Theriot C. M. Gulati A. S.. (2019). High-throughput amplicon sequencing of the full-length 16S rRNA gene with single-nucleotide resolution. Nucleic Acids Res. 47:e103. 31269198 10.1093/nar/gkz569 PMC6765137

[ref10] Cazemier A. E. Verdoes J. C. Reubsaet F. A. G. Hackstein J. H. P. van der Drift C. Op den Camp H. J. (2003). *Promicromonospora pachnodae* sp. nov., a member of the (hemi)cellulolytic hindgut flora of larvae of the scarab beetle *Pachnoda marginata*. Antonie Van Leeuwenhoek 83, 135–148. doi: 10.1023/A:1023325817663 12785307

[ref11] Cazemier A. E. Verdoes J. C. Van Ooyen A. J. J. Op den Camp H. J. M. (1999). Molecular and biochemical characterization of two xylanase-encoding genes from *Cellulomonas pachnodae*. Appl. Environ. Microbiol. 65, 4099–4107. doi: 10.1128/AEM.65.9.4099-4107.1999, PMID: 10473422 PMC99747

[ref12] Chen S. He Q. (2015). Persistence of Methanosaeta populations in anaerobic digestion during process instability. J. Ind. Microbiol. Biotechnol. 42, 1129–1137. doi: 10.1007/s10295-015-1632-7, PMID: 25956380

[ref13] Chen R. Li Z. Feng J. Zhao L. Yu J. (2020). Effects of digestate recirculation ratios on biogas production and methane yield of continuous dry anaerobic digestion. Bioresour. Technol. 316:123963. doi: 10.1016/j.biortech.2020.123963, PMID: 32795872

[ref001] Chevrette M. G. Carlson C. M. Ortega H. E. Thomas C. Ananiev G. E. Barns K. J.. (2019). The antimicrobial potential of Streptomyces from insect microbiomes. Nat. Commun. 10:516. 30705269 10.1038/s41467-019-08438-0 PMC6355912

[ref14] Chouaia B. Goda N. Mazza G. Alali S. Florian F. Gionechetti F.. (2019). Developmental stages and gut microenvironments influence gut microbiota dynamics in the invasive beetle *Popillia japonica* Newman (Coleoptera: Scarabaeidae). Environ. Microbiol. 21, 4343–4359. doi: 10.1111/1462-2920.14797, PMID: 31502415

[ref15] Collyer M. Adams D. (2021). RRPP: Linear model evaluation with randomized residuals in a permutation procedure. Vienna, Austria: R Foundation for Statistical Computing.

[ref16] De Cáceres M. Legendre P. (2009). Associations between species and groups of sites: indices and statistical inference. Ecology 90, 3566–3574. doi: 10.1890/08-1823.1 20120823

[ref17] De Cáceres M. Legendre P. Wiser S. K. Brotons L. (2012). Using species combinations in indicator value analyses. Methods Ecol. Evol. 3, 973–982. doi: 10.1111/j.2041-210X.2012.00246.x

[ref18] Dedysh S. N. Kulichevskaya I. S. Beletsky A. V. Ivanova A. A. Rijpstra W. I. C. Damsté J. S. S.. (2020). *Lacipirellula parvula* gen. nov., sp. nov., representing a lineage of planctomycetes widespread in low-oxygen habitats, description of the family Lacipirellulaceae fam. nov. and proposal of the orders Pirellulales ord. nov., Gemmatales ord. nov. and Isosphaerales ord. nov. Syst. Appl. Microbiol. 43:126050. doi: 10.1016/j.syapm.2019.126050 31882205 PMC6995999

[ref19] Donaldson G. P. Lee S. M. Mazmanian S. K. (2016). Gut biogeography of the bacterial microbiota. Nat. Rev. Microbiol. 14, 20–32. doi: 10.1038/nrmicro3552, PMID: 26499895 PMC4837114

[ref20] Ebert K. M. Arnold W. G. Ebert P. R. Merritt D. J. (2021). Hindgut microbiota reflects different digestive strategies in dung beetles (Coleoptera: Scarabaeidae: Scarabaeinae). Appl. Environ. Microbiol. 87, e02100–e02120. doi: 10.1128/AEM.02100-20 33355113 PMC8090880

[ref21] Egert M. Stingl U. Bruun L. D. Pommerenke B. Brune A. Friedrich M. W. (2005). Structure and topology of microbial communities in the major gut compartments of *Melolontha* larvae (Coleoptera: Scarabaeidae). Appl. Environ. Microbiol. 71, 4556–4566. doi: 10.1128/AEM.71.8.4556-4566.2005, PMID: 16085849 PMC1183286

[ref22] Egert M. Wagner B. Lemke T. Brune A. Friedrich M. W. (2003). Microbial community structure in midgut and hindgut of the humus-feeding larva of *Pachnoda ephippiata* (Coleoptera: Scarabaeidae). Appl. Environ. Microbiol. 69, 6659–6668. doi: 10.1128/AEM.69.11.6659-6668.2003, PMID: 14602626 PMC262301

[ref23] Engel P. Moran N. A. (2013). The gut microbiota of insects – diversity in structure and function. FEMS Microbiol. Rev. 37, 699–735. doi: 10.1111/1574-6976.12025 23692388

[ref24] Enzmann F. Mayer F. Rother M. Holtmann D. (2018). Methanogens: biochemical background and biotechnological applications. AMB Express 8:1. doi: 10.1186/s13568-017-0531-x, PMID: 29302756 PMC5754280

[ref25] Estes A. M. Hearn D. J. Snell-Rood E. C. Feindler M. Feeser K. Abebe T.. (2013). Brood ball-mediated transmission of microbiome members in the dung beetle, *Onthophagus taurus* (Coleoptera: Scarabaeidae). PLoS One 8:e79061. doi: 10.1371/journal.pone.0079061, PMID: 24223880 PMC3815100

[ref26] Goodrich J. K. Waters J. L. Poole A. C. Sutter J. L. Koren O. Blekhman R.. (2014). Human genetics shape the gut microbiome. Cells 159, 789–799. doi: 10.1016/j.cell.2014.09.053 PMC4255478 25417156

[ref27] Hammer T. J. Moran N. A. (2019). Links between metamorphosis and symbiosis in holometabolous insects. Philos. Trans. R. Soc. Lond. Ser. B Biol. Sci. 374:20190068. doi: 10.1098/rstb.2019.0068, PMID: 31438811 PMC6711286

[ref28] Hammons D. Kurtural S. Potter D. (2008). Japanese beetles facilitate feeding by green June beetles (Coleoptera: Scarabaeidae) on ripening grapes. Environ. Entomol. 37, 608–614. doi: 10.1603/0046-225x(2008)37[608:jbffbg]2.0.co;2 18419935

[ref29] Handique G. Phukan A. Bhattacharyya B. Baruah A. A. Rahman S. W. Baruah R. (2017). Characterization of cellulose degrading bacteria from the larval gut of the white grub beetle *Lepidiota mansueta* (Coleoptera: Scarabaeidae). Arch. Insect Biochem. Physiol. 94:e21370. doi: 10.1002/arch.21370, PMID: 28094878

[ref30] Hester J. Csárdi G. Wickham H. Chang W. RStudio Morgan M.. (2021). Remotes: R Package Installation from Remote Repositories, Including “GitHub.” Available at: https://cran.r-project.org/package=remotes

[ref32] Hobbie S. Li X. Basen M. Stingl U. Brune A. (2012). Humic substance-mediated Fe(III) reduction by a fermenting *Bacillus* strain from the alkaline gut of a humus-feeding scarab beetle larva. Syst. Appl. Microbiol. 35, 226–232. doi: 10.1016/j.syapm.2012.03.003, PMID: 22525666

[ref33] Hofstad T. Olsen I. Eribe E. R. Falsen E. Collins M. D. Lawson P. A. (2000). Dysgonomonas gen. nov. to accommodate *Dysgonomonas gadei* sp. nov., an organism isolated from a human gall bladder, and *Dysgonomonas capnocytophagoides* (formerly CDC group DF-3). Int. J. Syst. Evol. Microbiol. 50 Pt 6, 2189–2195. doi: 10.1099/00207713-50-6-2189, PMID: 11155996

[ref010] Holman D. B. Kommadath A. Tingley J. P. Abbott D. W. (2022). Novel Insights into the Pig Gut Microbiome Using Metagenome-Assembled Genomes. Microbiol Spectr. 10:e0238022. 35880887 10.1128/spectrum.02380-22 PMC9431278

[ref34] Hong S. Sun Y. Sun D. Wang C. (2022). Microbiome assembly on *Drosophila* body surfaces benefits the flies to combat fungal infections. iScience 25:104408. doi: 10.1016/j.isci.2022.104408, PMID: 35663020 PMC9157200

[ref37] Huang Q. Lopez D. Evans J. D. (2019). Shared and unique microbes between small hive beetles (*Aethina tumida*) and their honey bee hosts. Microbiology 8:e899. doi: 10.1002/mbo3.899 PMC6813432 31271530

[ref38] Huang S. Zhang H. (2013). The impact of environmental heterogeneity and life stage on the hindgut microbiota of *Holotrichia parallela* larvae (Coleoptera: Scarabaeidae). PLoS One 8:e57169. doi: 10.1371/journal.pone.0057169, PMID: 23437336 PMC3578786

[ref40] Jing T. Z. Qi F. H. Wang Z. Y. (2020). Most dominant roles of insect gut bacteria: digestion, detoxification, or essential nutrient provision? Microbiome 8:38. doi: 10.1186/s40168-020-00823-y, PMID: 32178739 PMC7077154

[ref41] Johnson D. T. Vishniac H. S. (1991). The role of *Trichosporon cutaneum* in eliciting aggregation behavior in *Cotinis nitida*. (Coleoptera: Scarabeidae). Environ. Entomol. 20, 15–21.

[ref42] Jose P. A. Ben-Yosef M. Lahuatte P. Causton C. E. Heimpel G. E. Jurkevitch E.. (2021). Shifting microbiomes complement life stage transitions and diet of the bird parasite *Philornis downsi* from the Galapagos Islands. Environ. Microbiol. 23, 5014–5029. doi: 10.1111/1462-2920.15435, PMID: 33587780

[ref008] Katoh K. Misawa K. Kuma K. Miyata T. (2002). MAFFT: a novel method for rapid multiple sequence alignment based on fast Fourier transform. Nucleic Acids Res. 30, 3059–3066. 12136088 10.1093/nar/gkf436 PMC135756

[ref43] Kikuchi Y. Hayatsu M. Hosokawa T. Nagayama A. Tago K. Fukatsu T. (2012). Symbiont-mediated insecticide resistance. Proc. Natl. Acad. Sci. U. S. A. 109, 8618–8622. doi: 10.1073/pnas.1200231109, PMID: 22529384 PMC3365206

[ref44] Kozich J. J. Westcott S. L. Baxter N. T. Highlander S. K. Schloss P. D. (2013). Development of a dual-index sequencing strategy and curation pipeline for analyzing amplicon sequence data on the MiSeq Illumina sequencing platform. Appl. Environ. Microbiol. 79, 5112–5120. doi: 10.1128/AEM.01043-13, PMID: 23793624 PMC3753973

[ref45] Kropp C. Le Corf K. Relizani K. Tambosco K. Martinez C. Chain F.. (2021). The keystone commensal bacterium *Christensenella minuta* DSM 22607 displays anti-inflammatory properties both in vitro and in vivo. Sci. Rep. 11:11494. doi: 10.1038/s41598-021-90885-1, PMID: 34075098 PMC8169850

[ref46] Kuo C. H. Huang P. Y. Sheu S. Y. Sheu D. S. Jheng L. C. Chen W. M. (2021). Zophobihabitans entericus gen. Nov., sp. nov., a new member of the family Orbaceae isolated from the gut of a superworm Zophobas morio. Int. J. Syst. Evol. Microbiol. 71:005081. doi: 10.1099/ijsem.0.005081, PMID: 34748472

[ref47] La Reau A. J. Suen G. (2018). The Ruminococci: key symbionts of the gut ecosystem. J. Microbiol. 56, 199–208. doi: 10.1007/s12275-018-8024-4, PMID: 29492877

[ref48] Lampert N. Mikaelyan A. Brune A. (2019). Diet is not the primary driver of bacterial community structure in the gut of litter-feeding cockroaches. BMC Microbiol. 19:238. doi: 10.1186/s12866-019-1601-9, PMID: 31666028 PMC6864750

[ref49] Lozupone C. Lladser M. E. Knights D. Stombaugh J. Knight R. (2011). UNiFrac: an effective distance metric for microbial community comparison. ISME J. 5, 169–172. doi: 10.1038/ismej.2010.133, PMID: 20827291 PMC3105689

[ref50] Lyon N. J. (2021). Gut bacteria of adult and larval *Cotinis nitida* Linnaeus (Coleoptera: Scarabaeidae) demonstrate community differences according to life stage and gut region: tidy, analysis, and plotting code. Available at: https://github.com/NJLyon-projects/Kucuk-Cotinis-Collab 10.3389/fmicb.2023.1185661 PMC10362445 37485511

[ref51] Lyon N. J. (2023). supportR: support functions for wrangling and visualization. R package version 1.0.0.

[ref52] Maire J. Vincent-Monégat C. Masson F. Zaidman-Rémy A. Heddi A. (2018). An IMD-like pathway mediates both endosymbiont control and host immunity in the cereal weevil Sitophilus spp. Microbiome. 6:6. doi: 10.1186/s40168-017-0397-9, PMID: 29310713 PMC5759881

[ref54] McKenna D. D. Shin S. Ahrens D. Balke M. Beza-Beza C. Clarke D. J.. (2019). The evolution and genomic basis of beetle diversity. Proc. Natl. Acad. Sci. U. S. A. 116, 24729–24737. doi: 10.1073/pnas.1909655116, PMID: 31740605 PMC6900523

[ref55] McManus R. Ravenscraft A. Moore W. (2018). Bacterial associates of a gregarious riparian beetle with explosive defensive chemistry. Front. Microbiol. 9:2361. doi: 10.3389/fmicb.2018.02361, PMID: 30344514 PMC6182187

[ref56] Mikaelyan A. Thompson C. L. Hofer M. J. Brune A. (2016). Deterministic assembly of complex bacterial communities in guts of germ-free cockroaches. Appl. Environ. Microbiol. 82, 1256–1263. doi: 10.1128/AEM.03700-15, PMID: 26655763 PMC4751828

[ref57] Oksanen J. Blanchet F. G. Friendly M. Kindt R. Legendre P. McGlinn D.. (2020). vegan: Community Ecology Package. Available at: https://CRAN.R-project.org/package=vegan

[ref58] Paradis E. Claude J. Strimmer K. (2004). APE: analyses of phylogenetics and evolution in R language. Bioinformatics 20, 289–290. doi: 10.1093/bioinformatics/btg412, PMID: 14734327

[ref59] Parker B. J. Wearsch P. A. Veloo A. C. M. Rodriguez-Palacios A. (2020). The genus Alistipes: gut Bacteria with emerging implications to inflammation, Cancer, and mental health. Front. Immunol. 11:906. doi: 10.3389/fimmu.2020.00906, PMID: 32582143 PMC7296073

[ref60] Potter D. A. (1991). Ecology and management of turfgrass insects. Annu. Rev. Entomol. 36, 383–406. doi: 10.1146/annurev.en.36.010191.002123

[ref61] Pramono A. K. Sakamoto M. Iino T. Hongoh Y. Ohkuma M. (2015). *Dysgonomonas termitidis* sp. nov., isolated from the gut of the subterranean termite *Reticulitermes speratus*. Int. J. Syst. Evol. Microbiol. 65, 681–685. doi: 10.1099/ijs.0.070391-0 25428419

[ref62] Price M. N. Dehal P. S. Arkin A. P. (2010). FastTree 2–approximately maximum-likelihood trees for large alignments. PLoS One 5:e9490. doi: 10.1371/journal.pone.0009490, PMID: 20224823 PMC2835736

[ref002] Qadri M. Short S. Gast K. Hernandez J. Wong A. C.-N. (2020). Microbiome innovation in agriculture: development of microbial based tools for insect pest management. Front. Sustain. Food Syst. 4.

[ref63] Quast C. Pruesse E. Yilmaz P. Gerken J. Schweer T. Yarza P.. (2013). The SILVA ribosomal RNA gene database project: improved data processing and web-based tools. Nucleic Acids Res. 41, D590–D596. doi: 10.1093/nar/gks1219, PMID: 23193283 PMC3531112

[ref011] R Core Team (2020). R: A language and environment for statistical computing. R Foundation for Statistical Computing, Vienna, Austria. Available at: https://www.R-project.org/.

[ref012] Rey F. E. Gonzalez M. D. Cheng J. Wu M. Ahern P. P. Gordon J. I. (2013). Metabolic niche of a prominent sulfate-reducing human gut bacterium. Proc. Natl. Acad. Sci. U S A. 110, 13582–13587. 23898195 10.1073/pnas.1312524110 PMC3746858

[ref64] Schnorr S. L. Hofman C. A. Netshifhefhe S. R. Duncan F. D. Honap T. P. Lesnik J.. (2019). Taxonomic features and comparisons of the gut microbiome from two edible fungus-farming termites (*Macrotermes falciger*; *M. natalensis*) harvested in the Vhembe district of Limpopo, South Africa. BMC Microbiol. 19:164. doi: 10.1186/s12866-019-1540-5, PMID: 31315576 PMC6637627

[ref65] Schroeder B. G. Logroño W. Rocha U. N. D. Harms H. Nikolausz M. (2022). Enrichment of anaerobic microbial communities from Midgut and hindgut of Sun beetle larvae (*Pachnoda marginata*) on wheat straw: effect of inoculum preparation. Microorganisms. 10:761. doi: 10.3390/microorganisms10040761, PMID: 35456811 PMC9024811

[ref66] Schwab D. B. Riggs H. E. Newton I. L. Moczek A. P. (2016). Developmental and ecological benefits of the maternally transmitted microbiota in a dung beetle. Am. Nat. 188, 679–692. doi: 10.1086/688926, PMID: 27860508

[ref67] Shukla S. P. Sanders J. G. Byrne M. J. Pierce N. E. (2016). Gut microbiota of dung beetles correspond to dietary specializations of adults and larvae. Mol. Ecol. 25, 6092–6106. doi: 10.1111/mec.13901 27801992

[ref69] Suárez-Moo P. Cruz-Rosales M. Ibarra-Laclette E. Desgarennes D. Huerta C. Lamelas A. (2020). Diversity and composition of the gut microbiota in the developmental stages of the dung beetle *Copris incertus* say (Coleoptera, Scarabaeidae). Front. Microbiol. 11:1698. doi: 10.3389/fmicb.2020.01698 32793162 PMC7393143

[ref013] Torvalds L. Hamano J. (2021). Git – fast, scalable, distributed revision control system. Available at: https://git-scm.com/

[ref70] Vavrek M. J. (2020). Fossil: Palaeoecological and Palaeogeographical analysis tools. Palaeontologia Electronica. 14:16.

[ref71] Vishniac H. Johnson D. (1990). Development of a yeast Flora in the adult green June beetle (*Cotinis nitida*). Mycologia 82, 471–479. doi: 10.1080/00275514.1990.12025910

[ref72] Wang K. Gao P. Geng L. Liu C. Zhang J. Shu C. (2022). Lignocellulose degradation in *Protaetia brevitarsis* larvae digestive tract: refining on a tightly designed microbial fermentation production line. Microbiome. 10:90. doi: 10.1186/s40168-022-01291-2, PMID: 35698170 PMC9195238

[ref73] Wang Y. Rozen D. E. (2017). Gut microbiota colonization and transmission in the burying beetle *Nicrophorus vespilloides* throughout development. Appl. Environ. Microbiol. 83, e03250–e03216. 28213538 10.1128/AEM.03250-16 PMC5394326

[ref74] Wang M. Xiang X. Wan X. (2020). Divergence in gut bacterial community among life stages of the rainbow stag beetle *Phalacrognathus muelleri* (Coleoptera: Lucanidae). Insects. 11:719. doi: 10.3390/insects11100719, PMID: 33096611 PMC7589407

[ref75] Whitcomb R. F. Vignault J.-C. Tully J. G. Rose D. L. Carle P. Bove J. M.. (1993). Spiroplasma clarkii sp. nov. from the Green June Beetle (Coleoptera: Scarabaeidae). Int. J. Syst. Bacteriol. 43, 261–265.

[ref76] Wickham H. (2016). ggplot2: elegant graphics for data analysis. Available at: https://ggplot2.tidyverse.org

[ref77] Wickham H. RStudio. (2019). Stringr: simple, Consistent Wrappers for Common String Operations. Available at: https://CRAN.R-project.org/package=stringr

[ref78] Wickham H. RStudio. (2021). Tidyverse: Easily Install and Load the “Tidyverse.” Available at: https://cran.r-project.org/package=tidyverse

[ref79] Willms I. M. Rudolph A. Y. Göschel I. Bolz S. H. Schneider D. Penone C.. (2020). Globally abundant "Candidatus Udaeobacter" benefits from release of antibiotics in soil and potentially performs trace gas scavenging. mSphere 5:e00186-20. doi: 10.1128/mSphere.00186-20 32641424 PMC7343977

[ref81] Xie J. Butler S. Sanchez G. Mateos M. (2014). Male killing Spiroplasma protects *Drosophila melanogaster* against two parasitoid wasps. Heredity (Edinb). 112, 399–408. doi: 10.1038/hdy.2013.118, PMID: 24281548 PMC3966124

[ref82] Zheng H. Nishida A. Kwong W. K. Koch H. Engel P. Steele M. I.. (2016). Metabolism of toxic sugars by strains of the bee gut Symbiont *Gilliamella apicola*. MBio 7, e01326–e01316. doi: 10.1128/mBio.01326-16 27803186 PMC5090037

