# Supplementary material for: Gut bacteria of adult and larval Cotinis nitida Linnaeus (Coleoptera: Scarabaeidae) demonstrate community differences according to respective life stage and gut region
Source: Front Microbiol. 2023 Jul 7;14:1185661. doi: 10.3389/fmicb.2023.1185661 (PMC10362445; doi:10.3389/fmicb.2023.1185661)
Supplement: Supplementary file 1 [file Data_Sheet_1.PDF]

Sample ID	Lifestage	Gut Region	Sex	Filtered reads	Denoised reads	ASV count
All Samples	N/A	N/A	N/A	777,901	569,124	3,622
Amid12	adult	midgut	male	3,373	2,834	51
Amid39	adult	midgut	female	2,954	2,251	25
Amid40	adult	midgut	male	5,773	4,505	31
Amid41	adult	midgut	male	2,550	1,949	13
Amid46	adult	midgut	male	4,539	3,567	26
Amid48	adult	midgut	female	409	346	15
Amid49	adult	midgut	female	6,504	5,251	49
Amid7	adult	midgut	male	1,433	1,118	31
hind12	adult	hindgut	male	19,943	15,704	86
hind39	adult	hindgut	female	22,202	16,881	71
hind4	adult	hindgut	female	36,020	29,124	108
hind40	adult	hindgut	male	35,151	28,052	137
hind41	adult	hindgut	male	26,015	21,089	139
hind46	adult	hindgut	female	29,546	23,740	125
hind48	adult	hindgut	female	25,170	19,718	113
hind49	adult	hindgut	female	16,697	13,636	126
hind7	adult	hindgut	male	26,561	20,485	87
Ileum12	larva	ileum	N/A	9,215	6,830	345
Ileum17	larva	ileum	N/A	15,281	10,720	304
Ileum18	larva	ileum	N/A	7,369	5,812	98
Ileum19	larva	ileum	N/A	7,944	6,440	72
Ileum2	larva	ileum	N/A	9,239	5,524	668
Ileum20	larva	ileum	N/A	3,514	2,353	189
Ileum21	larva	ileum	N/A	5,061	3,491	131
Ileum3	larva	ileum	N/A	22,905	13,426	1,045
Ileum5	larva	ileum	N/A	30,525	19,726	1,061
Ileum7	larva	ileum	N/A	10,399	6,205	700
Ileum9	larva	ileum	N/A	35,260	20,867	1,234
Lmid2	larva	midgut	N/A	9,419	4,887	747
Lmid3	larva	midgut	N/A	11,029	5,817	845
Lmid5	larva	midgut	N/A	2,074	1,214	266
Lmid9	larva	midgut	N/A	12,713	6,246	839
Paunch12	larva	paunch	N/A	29,244	21,892	848
Paunch17	larva	paunch	N/A	35,366	26,009	831
Paunch18	larva	paunch	N/A	41,880	30,281	984
Paunch19	larva	paunch	N/A	38,719	28,897	770
Paunch2	larva	paunch	N/A	30,647	23,035	1,150
Paunch20	larva	paunch	N/A	30,541	22,872	940
Paunch21	larva	paunch	N/A	16,744	12,413	443

Paunch3	larva	paunch	N/A	48,987	37,689	1,358
Paunch5	larva	paunch	N/A	26,032	19,817	1,060
Paunch9	larva	paunch	N/A	22,954	16,411	812
