# Supplementary figures and images for: Gut bacteria of adult and larval Cotinis nitida Linnaeus (Coleoptera: Scarabaeidae) demonstrate community differences according to respective life stage and gut region
Source: Front Microbiol. 2023 Jul 7;14:1185661. doi: 10.3389/fmicb.2023.1185661 (PMC10362445; doi:10.3389/fmicb.2023.1185661)

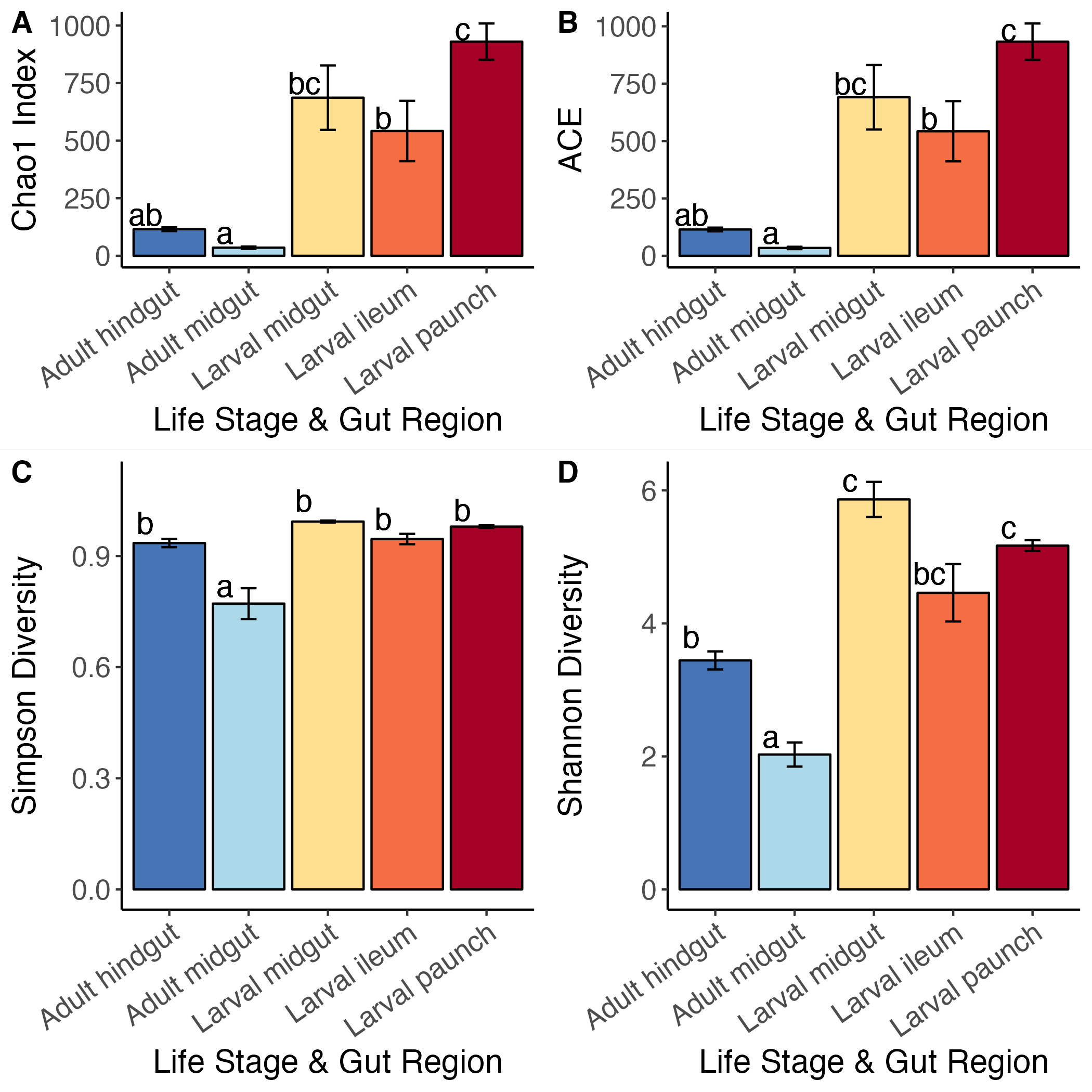

Supplement: Supplementary file 2 [file Image_1.TIFF]

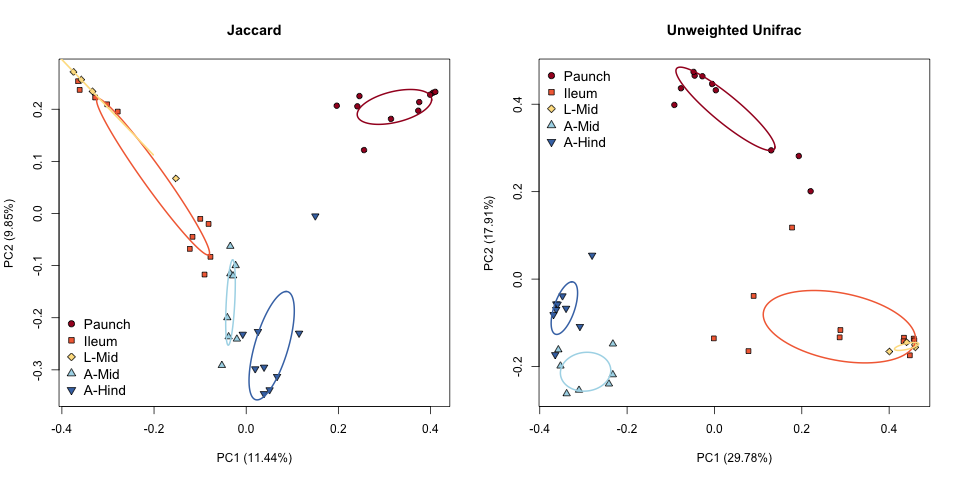

Supplement: Supplementary file 3 [file Image_2.TIFF]
